# Cerebrospinal fluid findings in patients with myelin oligodendrocyte glycoprotein (MOG) antibodies. Part 2: Results from 108 lumbar punctures in 80 pediatric patients

**DOI:** 10.1186/s12974-020-01825-1

**Published:** 2020-09-03

**Authors:** Sven Jarius, Christian Lechner, Eva M. Wendel, Matthias Baumann, Markus Breu, Mareike Schimmel, Michael Karenfort, Adela Della Marina, Andreas Merkenschlager, Charlotte Thiels, Astrid Blaschek, Michela Salandin, Steffen Leiz, Frank Leypoldt, Alexander Pschibul, Annette Hackenberg, Andreas Hahn, Steffen Syrbe, Jurgis Strautmanis, Martin Häusler, Peter Krieg, Astrid Eisenkölbl, Johannes Stoffels, Matthias Eckenweiler, Ilya Ayzenberg, Jürgen Haas, Romana Höftberger, Ingo Kleiter, Mirjam Korporal-Kuhnke, Marius Ringelstein, Klemens Ruprecht, Nadja Siebert, Kathrin Schanda, Orhan Aktas, Friedemann Paul, Markus Reindl, Brigitte Wildemann, Kevin Rostásy, in cooperation with the BIOMARKER study group and the Neuromyelitis optica Study Group (NEMOS)

**Affiliations:** 1 Molecular Neuroimmunology Group, Department of Neurology, University of Heidelberg, Heidelberg, Germany; 2 Division of Pediatric Neurology, Department of Pediatrics I, Medical University of Innsbruck, Innsbruck, Austria; 3 Department of Pediatrics, Olgahospital, Klinikum Stuttgart, Stuttgart, Germany; 4 Department of Pediatric and Adolescent Medicine, Medical University of Vienna, Vienna, Austria; 5 Division of Pediatric Neurology, Children’s Hospital, Medical University of Augsburg, Augsburg, Germany; 6 Department of General Pediatrics, Neonatology and Pediatric Cardiology, University Children’s Hospital, Heinrich-Heine-University, Düsseldorf, Germany; 7 Department of Neuropediatrics, Developmental Neurology and Social Pediatrics, Children’s Hospital, University of Duisburg-Essen, Duisburg, Germany; 8 Division of Pediatric Neurology, University Hospital for Children and Adolescents, Leipzig, Germany; 9 Department of Neuropediatrics, University Children’s Hospital, Ruhr-University Bochum, Bochum, Germany; 10 Department of Pediatric Neurology and Developmental Medicine, Dr. von Hauner Children’s Hospital, University of Munich, Munich, Germany; 11 Department of Pediatrics, Bozen Hospital, Bozen, Italy; 12 Department of Pediatrics, Division of Pediatric Neurology, Klinikum Dritter Orden, Munich, Germany; 13 Neuroimmunology, Institute of Clinical Chemistry and Department of Neurology, Christian-Albrechts-University Kiel and Medical University Hospital Schleswig-Holstein, Kiel, Germany; 14 Department of Neuropediatrics and Muscle Disorders, University Medical Center, Faculty of Medicine, University of Freiburg, Freiburg, Germany; 15 Division of Pediatric Neurology, University Children’s Hospital Zurich, Zurich, Switzerland; 16 Department of Pediatric Neurology, University Children’s Hospital Giessen, Giessen, Germany; 17 Division of Child Neurology and Inherited Metabolic Diseases, Department of General Pediatrics, Center for Child and Adolescent Medicine, Heidelberg University Hospital, Heidelberg, Germany; 18 Department of Neurology, Children’s Clinical University Hospital, Riga, Latvia; 19 Department of Pediatrics, Division of Neuropediatrics and Social Pediatrics, Medical University RWTH Aachen, Aachen, Germany; 20 Department of Pediatrics, Städtisches Klinikum Karlsruhe, Karlsruhe, Germany; 21 Department of Pediatrics, Women’s and Children’s Hospital, Linz, Austria; 22 Department of Pediatric Neurology, Children’s Hospital Neuburg, Neuburg, Germany; 23 Department of Neurology, St Josef Hospital, Ruhr-University Bochum, Bochum, Germany; 24 Institute of Neurology, Medical University of Vienna, Vienna, Austria; 25 Marianne-Strauß-Klinik, Behandlungszentrum Kempfenhausen für Multiple Sklerose Kranke gGmbH, Berg, Germany; 26 Department of Neurology, Medical Faculty, Heinrich Heine University Dusseldorf, Düsseldorf, Germany; 27 Department of Neurology, Center for Neurology and Neuropsychiatry, LVR-Klinikum, Heinrich Heine University Dusseldorf, Düsseldorf, Germany; 28 Department of Neurology, Charité - Universitätsmedizin Berlin, Berlin, Germany; 29 NeuroCure Clinical Research Center, Charité - Universitätsmedizin Berlin, Berlin, Germany; 30 Experimental and Clinical Research Center, Max Delbrueck Center for Molecular Medicine, and Charité Universitätsmedizin Berlin, Berlin, Germany; 31 Clinical Department of Neurology, Medical University of Innsbruck, Innsbruck, Austria; 32 Department of Pediatric Neurology, Children’s Hospital Datteln, University Witten/Herdecke, Datteln, Germany

**Keywords:** MOG antibody-associated disease (MOGAD), Myelin oligodendrocyte glycoprotein (MOG), Antibodies, Encephalomyelitis, Cerebrospinal fluid, Lumbar puncture, Optic neuritis, Transverse myelitis, Neuromyelitis optica (Devic syndrome), NMO spectrum disorders, Brainstem encephalitis, Acute disseminated encephalomyelitis (ADEM), Children, Multiple sclerosis (MS), Oligoclonal bands

## Abstract

**Background:**

New-generation, cell-based assays have demonstrated a robust association of serum autoantibodies to full-length human myelin oligodendrocyte glycoprotein (MOG-IgG) with (mostly recurrent) optic neuritis, myelitis, and brainstem encephalitis, as well as with neuromyelitis optica (NMO)-like or acute-disseminated encephalomyelitis (ADEM)-like presentations. However, only limited data are yet available on cerebrospinal fluid (CSF) findings in MOG-IgG-associated encephalomyelitis (MOG-EM; also termed MOG antibody-associated disease, MOGAD).

**Objective:**

To describe systematically the CSF profile in children with MOG-EM.

**Material and methods:**

Cytological and biochemical findings (including white cell counts [WCC] and differentiation; frequency and patterns of oligoclonal bands; IgG/IgM/IgA and albumin concentrations and CSF/serum ratios; intrathecal IgG/IgM/IgA fractions; locally produced IgG/IgM/IgA concentrations; immunoglobulin class patterns; IgG/IgA/IgM reibergrams; Link index; measles/rubella/zoster [MRZ] reaction; other anti-viral and anti-bacterial antibody indices; CSF total protein; CSF l-lactate) from 108 lumbar punctures in 80 pediatric patients of mainly Caucasian descent with MOG-EM were analyzed retrospectively.

**Results:**

Most strikingly, CSF-restricted oligoclonal IgG bands, a hallmark of multiple sclerosis (MS), were absent in 89% of samples (*N* = 96), and the MRZ reaction, the most specific laboratory marker of MS known so far, in 100% (*N* = 29). If present at all, intrathecal IgG synthesis was low, often transient and mostly restricted to acute attacks. Intrathecal IgM synthesis was present in 21% and exclusively detectable during acute attacks. CSF WCC were elevated in 54% of samples (median 40 cells/μl; range 6–256; mostly lymphocytes and monocytes; > 100/μl in 11%). Neutrophils were present in 71% of samples; eosinophils, activated lymphocytes, and plasma cells were seen only rarely (all < 7%). Blood–CSF barrier dysfunction (as indicated by an elevated albumin CSF/serum ratio) was present in 46% of all samples (*N* = 79) and at least once in 48% of all patients (*N* = 67) tested. CSF alterations were significantly more frequent and/or more pronounced in patients with acute spinal cord or brain disease than in patients with acute ON and varied strongly depending on attack severity. CSF l-lactate levels correlated significantly with the spinal cord lesions load (measured in vertebral segments) in patients with acute myelitis (*p* = 0.0099). An analysis of pooled data from the pediatric and the adult cohort showed a significant relationship of QAlb (*p* < 0.0005), CST TP (*p* < 0.0001), and CSF l-lactate (*p* < 0.0003) during acute attacks with age.

**Conclusion:**

MOG-IgG-associated EM in children is characterized by CSF features that are distinct from those in MS. With regard to most parameters, no marked differences between the pediatric cohort and the adult cohort analyzed in Part 1 were noted. Our findings are important for the differential diagnosis of pediatric MS and MOG-EM and add to the understanding of the immunopathogenesis of this newly described autoimmune disease.

## Introduction

Over the past few years, several studies using new-generation cell-based assays (CBA) have demonstrated a robust association of immunoglobulin G (IgG) autoantibodies targeting full-length, conformationally intact human myelin oligodendrocyte glycoprotein (MOG) with (mostly recurrent) optic neuritis (ON), myelitis, and brainstem encephalitis, as well as with neuromyelitis optica (NMO)-like and acute disseminated encephalomyelitis (ADEM)-like presentations, rather than with classic multiple sclerosis (MS) [1–11]. The suspected pathophysiological role of MOG-IgG was first described in children, who more often present with MOG-IgG-associated disorders than adults [12–15]. Based on evidence from (a) immunological studies suggesting a direct pathogenic impact of MOG-IgG, (b) neuropathological studies demonstrating discrete histopathological features, (c) serological studies reporting a lack of aquaporin-4 (AQP4)-IgG in almost all MOG-IgG-positive patients, and (d) cohort studies suggesting differences in clinical and paraclinical presentation, treatment response, and prognosis, MOG-IgG is now considered to denote a disease entity in its own right, distinct from classic MS and from AQP4-IgG-positive NMO spectrum disorders (NMOSD) [16–21], which is now often referred to as MOG-IgG-associated encephalomyelitis (MOG-EM) or MOG-IgG-associated autoimmune disease [11, 22, 23]. Several studies have shown that the proportion of patients with autoimmunity against MOG among all patients with inflammatory demyelinating CNS disorders is age-dependent with the highest seropositivity rates and highest MOG-IgG titers found in very young children [1, 24, 25]. ADEM-like disease is the predominant clinical presentation in young children, whereas in older children with MOG-IgG there is a shift toward ON, myelitis, and/or brainstem symptoms. MRI findings range from normal to widespread brain and spinal cord white and grey matter involvement [13].

So far, only limited data are available on the cerebrospinal fluid (CSF) profile in MOG-EM in pediatric patients. Previous studies were either based on relatively small patient numbers, included mainly adult patients, and/or did not consider Caucasian patients. Moreover, all investigated only a small number of selected CSF parameters.

In Part 1 of this article series, we report on the CSF findings in MOG-EM in adults [26]. For the present study, we systematically and comprehensively analyzed the results of 108 lumbar punctures (LP) from a cohort of 80 pediatric patients of mainly Caucasian descent with MOG-IgG-associated EM.

## Patients and methods

### Patients

Results from 108 lumbar punctures (LP) in 80 pediatric patients with MOG-EM were analyzed retrospectively. MOG-EM was defined as monophasic or relapsing acute ON, myelitis, brainstem encephalitis, or encephalitis associated with MRI or (in the case of ON only) electrophysiological findings compatible with CNS demyelination and with MOG-IgG as detected by means of a cell-based assay (CBA) employing human full-length MOG as antigen [27]. Longitudinally extensive transverse myelitis (LETM) was defined as acute myelitis with at least one contiguous lesion extending over three or more vertebral segments (VS) as detected by magnetic resonance imaging (MRI) [28, 29]. Cases of acute myelitis in which no lesion extended over more than two segments were classified as non-longitudinally extensive transverse myelitis (NETM). All patients were diagnosed with MOG-EM at German (Aachen, Augsburg, Berlin, Bochum, Datteln, Dusseldorf, Essen, Freiburg, Giessen, Göttingen, Heidelberg, Karlsruhe, Kiel/Lübeck, Leipzig, Munich, Neuburg, Oldenburg, Osnabrück, Vogtareuth), Austrian (Innsbruck, Linz, Vienna, Zams), Italian (Bozen), Latvian (Riga),and Swiss (Zurich) university hospitals and other tertiary care centers. All eligible patients seen at the respective centers were included. The participating centers are members of the BIOMARKER study group [13] and/or the Neuromyelitis Optica Study Group (NEMOS) [30]. All patients were tested for MOG-IgG by means of a CBA employing full-length human MOG as target antigen as recommended in the international consensus statement on MOG-IgG testing [27]. Assays used to detect MOG-IgG included three live CBA (Medical University Innsbruck, Austria; University of Vienna, Austria; Ludwig Maximilian University Munich, Germany) [1, 31–33], an in-house fixed CBA (University of Heidelberg, Germany) [2, 34], and a commercial fixed CBA (Euroimmun, Lübeck, Germany). Results from serial LP were available for 21/80 (26.3%) patients. In total, 28 follow-up CSF examinations were performed (median 1 follow-up sample per patient; range 1–3). The first LP was performed after a median of 2 days after disease onset and the follow-up LPs after a median of 12 days of the last attack; the proportion of samples taken during relapse did not significantly differ between the two groups (86% vs. 80%). The mean time interval between LPs was 256 days (median 52 days). None of the patients was positive for AQP4-IgG. The study was approved by the review boards of the participating centers. Patients gave written informed consent. LPs were performed for diagnostic purposes in all cases; no samples were obtained for this study.

### Evaluation of the humoral immune response

Oligoclonal IgG bands were assessed by isoelectric focusing and evaluated according to an international consensus [35]. Immunoglobulins and albumin were measured immunonephelometrically. Quantitative expressions of the intrathecal humoral immune response were based on calculation of the CSF/serum quotients QIgG, QIgM, and QIgA with *Q*_Ig_ = *I*g_CSF[mg/l]_/*I*g_serum[g/l]_. The upper limits of the respective reference ranges, *Q*_lim_(IgG), *Q*_lim_(IgM), and *Q*_lim_(IgA), were calculated against QAlb according to Reiber’s revised hyperbolic function [36]. Values for *Q*_Ig_ exceeding *Q*_lim_(Ig) were considered to indicate intrathecal immunoglobulin synthesis [36]. The fraction (in %) of intrathecally produced Ig (Ig_IF_) and the absolute amount of locally, i.e., intrathecally, produced Ig (IgG_loc_) were calculated according to the following formulas: Ig_IF[%]_ = [QIgG – Q_lim_(*I*g)] × *I*g_serum_ × 100 and *I*g_loc[mg/L]_ = [*Q*_Ig_ – *Q*_lim_(*I*g)] × *I*g_serum_, respectively [36]. CSF and serum concentrations for immunoglobulins and albumin, respectively, were analyzed within the same analytical series.

### Evaluation of the blood–CSF barrier

The CSF/serum albumin quotient, QAlb = Alb_CSF[mg/l]_/Alb_serum[g/l]_, was used to assess the blood–CSF barrier (BCB) function. As the upper reference limit of QAlb is age dependent, *Q*_lim_(Alb) was calculated as 4 + (*a* / 15) × 10^−3^ with *a* representing patient’s age according to Reiber et al. (1994) [37]. Dysfunction of the BCB was defined as QAlb > *Q*_lim_(Alb).

### Cytological examination, total CSF protein, and l-lactate

A white cell count > 5/μl was classified as increased [38]. An age-dependent reference range for CSF l-lactate was applied (0–15 years of age: 1.8 mmol/l, ≥ 16: 2.1 mmol/l) [38]. The upper reference limit for total CSF protein was set at 0.45 mg/l [38].

### Statistics

Samples were analyzed in total as well as after stratification according to disease status and treatment status. Fisher’s exact test, the Mann*–*Whitney *U* test, and the Kruskal*–*Wallis test were used to detect statistical differences between groups. Spearman’s rho was assessed to test for correlations. Due to the exploratory nature of this study, no correction for multiple testing was applied other than Dunn’s post-test. Reiber diagrams (reibergrams) were generated using *Protein Statistics in CSF analysis V3.0* software (Comed, Soest, Germany).

## Results

### Patient characteristics

The male:female ratio was 1:1.3. The median age at the time of LP was 6 years (range 0.6–17.7). A total of 99.1% of all samples were obtained from patients of Caucasian descent. The median disease duration was 0 months at the time of LP (maximum 118 months) and 34.5 months (range 0–229) at last follow-up. Information on the date of onset of the last attack prior to LP was available from the patient records for 105 samples. Of those, 94 (89.5%) were obtained within 45 days (median 2 days; range 0–44) after the onset of an acute attack (acute myelitis with or without other symptoms in 31.9% [“acute MY subgroup”]; acute ON but no myelitis in 28.7% [“acute ON subgroup”]; neither myelitis nor ON but isolated brain or brainstem/cerebellar disease in 39.4% [“acute BRAIN subgroup”]). Thirty-four of 37 samples (92%) samples in the acute BRAIN subgroup were obtained from patients who met the diagnostic criteria for ADEM [39] at the time of LP. Eleven samples were obtained more than 45 days after attack onset (“remission subgroup”). Of the 16.7% of samples that came from patients with acute myelitis and available MRI data, 25 (83.3%) were obtained during episodes of LETM. The cumulative spinal cord lesion load (summing up lesions in patients with multiple lesions) was six VS (up to 16 VS) in the total myelitis group and six VS in the LETM subgroup [28, 29]. Of the ON samples, 55.6% were taken during attacks of unilateral and 44.4% during attacks of bilateral ON. Attack severity was classified by the treating physicians as “mild” or “moderate” in 29% and as “severe” in 69.2% (missing data in the remainder). At last follow-up, 51.9% of all patients had experienced at least two attacks (“relapsing subgroup”) and 48.1% of patients had not relapsed (“monophasic subgroup”). The median disease duration at last follow-up was 63 months (range 1–229) in the relapsing subgroup and 21 months (range 0–65) in the monophasic subgroup. At first LP, 69/77 (89.6%) patients had been neither treated with steroids nor with immunosuppressants or immunomodulatory drugs (no precise data on the treatment status at the time of LP available for three patients). If all LPs are considered, 92/108 (85.2%) were obtained from patients who were untreated at the time of LP (information missing for 3 samples).

### Cellular immune response

An increased CSF white cell count (WCC) was found in 56/103 (54.4%) samples, which is almost exactly the rate found in adults [26], with a median of 40 cells/μl (range 6–256). WCC ≥ 50 cells/μl, which are very rare in MS (and thus considered a 'red flag' that should prompt physicians to challenge the diagnosis MS) [35, 40], were present in 21.4% (22/103) of samples and thus with virtually the same frequency as in adults. Marked pleocytosis, defined as CSF WCC ≥ 100 cells/μl, was found in 11/103 (10.7%) samples (median 179; range, 103–256), which compares to 12.1% in adults, all of which were taken during an acute attack in untreated patients. CSF WCC exceeded 200 cells/μl in only 3/103 (2.9%; vs. 4.5% in adults) samples and 300 cells/μl in no sample (vs. 1.9% in adults). In total, pleocytosis was noted at least once in 45/76 (59.2%) patients with available data (vs. 56.6% in adults).

As in adults, lymphocytes, which were found in 45/45 (100%) samples with available cytological data, and monocytes, detected in at least 35/45 (77.8%) samples, were the predominant immune cell types in the CSF. Relative lymphocyte counts ranged between 32 and 100% (median 90%; *N* = 37) of all CSF cells and relative monocyte counts between 4 and 39% (median 20%; *N* = 14).

Importantly, however, neutrophils were present in 71.1% (32/45) of samples (and thus even more frequent than in adults [43%; *p* < 0.003]). Neutrophils represented up to 69% of all leukocytes (data available for 28 samples) and up to 69% in samples with pleocytosis (*N* = 26). If only LPs with pleocytosis and available cytological data are considered, neutrophil granulocytes were present even in 88.2% (30/34) of samples (vs. 50% in adults). As in the adults, neutrophils were more commonly found during acute attacks in the MY and BRAIN subgroups (81% of all samples with available data) than in the ON subgroup (45%). The higher frequency of neutrophils in the pediatric cohort may thus reflect the higher proportion of ON attacks in the adult cohort (41% vs. 26% among samples with cytology data). In total, neutrophil granulocytes were present at least once in 29/38 (76.3%) patients with available cytology data (vs. 46% in adults).

By contrast, eosinophils and basophils were rare findings, present in only 3/45 (6.7%) (accounting for 20%, 2%, and a “high proportion” of all WCC; all reported in patients with pleocytosis [87, 21 and 179 cells/μl, respectively]) and 2/45 (4.4%) samples with available cytology data, respectively. Activated lymphocytes were noted in 3/45 (6.7%) samples, and plasma cells in 2/45 (4.4%). The results did not differ significantly from those in the adult cohort.

**Fig. 1:**
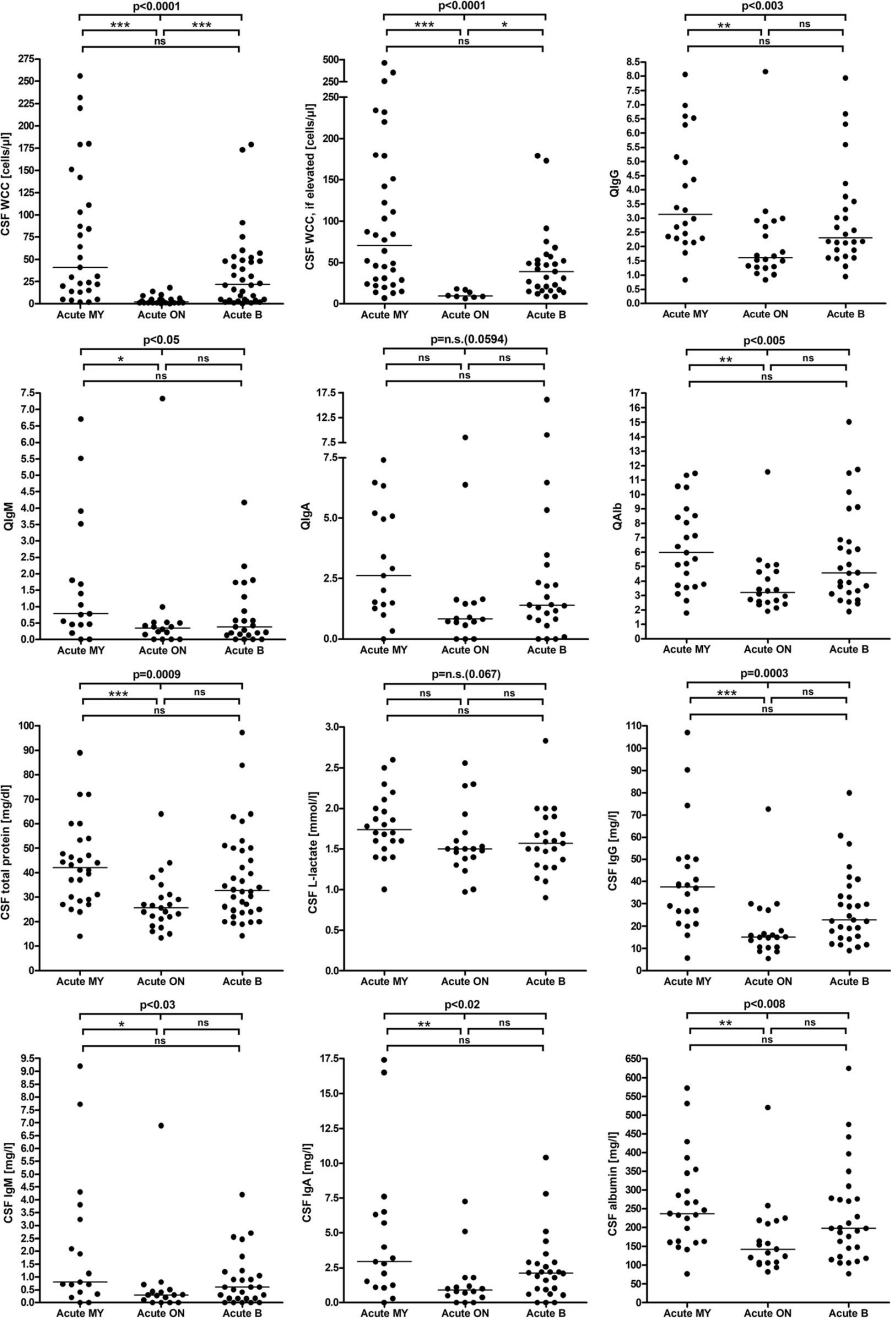
CSF white cell counts, IgG, IgA, IgM, and albumin CSF/serum ratios and CSF concentrations, CSF total protein concentrations, and CSF L-lactate concentrations in MOG-IgG-positive EM. A statistically significant difference between the acute MY subgroup and the acute ON subgroup was found regarding all parameters studied. *IgG*/*A*/*M* immunoglobulin G/A/M, *QIgG*/*A*/*M* CSF/serum IgG/A/M ratios, *QAlb* CSF/serum albumin ratio

Pleocytosis was significantly less common in the acute ON subgroup than in the acute myelitis (19.2% vs. 82.8%; *p* < 0.000003) and in the acute brain subgroup (19.2% vs. 66.7%; *p* < 0.0003). Similarly, median cell numbers were lower in the acute ON subgroup (2, range 0–18) than in the acute MY (41, range 2–256; *p* < 0.000001) and the acute brain subgroup (22, range 0–179; *p* < 0.000004) (Fig. 1). CSF WCC ≥ 50 cells/μl were found exclusively in patients with acute myelitis and to a lower extent in patients with acute brain disease at the time of LP but not in acute ON patients (present in 48.3%, or 14/29, in the acute MY subgroup, vs. 22.2%, or 8/36, in the acute brain subgroup, vs. 0%, or 0/26, in the acute ON subgroup). This is all highly similar to what was observed in the adult cohort [26]. See Table 1 and Supplementary Figure 1 for further details.

**Table 1:** CSF white cell counts (WCC) and cytology results in MOG-IgG-positive EM. WCC in the various subgroups are reported as medians; ranges and total sample numbers are given in brackets

	Units	Total	Attack	Remission	Acute MY subgroup	Acute ON subgroup	Acute BRAIN subgroup
CSF white cell counts							
Pleocytosis	Samples	56/103 (54.4%)	53/91 (58.2%)	3/11 (27.3%)	24/29 (82.8%)	5/26 (19.2%)	24/36 (66.7%)
WCC, all samples	Cells/μl	11.5 (0–256; 102)	14 (0–256; 91)	4 (0–35; 11)	41 (2–256; 29)	2 (0–18; 25)	22 (0–179; 36)
WCC, if elevated	Cells/μl	40 (6–256; 56)	42 (6–256; 53)	23 (18–35; 3)	70.5 (13–256; 24)	10 (6–18; 5)	44.5 (9–179; 24)
WCC, ≥ 100	Samples	11/103 (10.7%)	11/91 (12.1%)	0/11 (0%)	9/29 (31%)	0/26 (0%)	2/36 (5.6%)
WCC, ≥ 100	Cells/μl	179 (103–256; 11)	179 (103–256; 11)	n.a. (n.a.; 0)	179 (103–256; 9)	n.a. (n.a; 0)	176 (173–179; 2)
Lymphocytes	Samples	45/45 (100%)	43/43 (100%)	2/2 (100%)	20/20 (100%)	11/11 (100%)	12/12 (100%)
Monocytes	Samples	35/45 (77.8%)	34/43 (79.1%)	1/2 (50%)	15/20 (75%)	7/11 (63.6%)	12/12 (100%)
Neutrophils	Samples	32/45 (71.1%)	31/43 (72.1%)	1/2 (50%)	15/20 (75%)	5/11 (45.5%)	11/12 (91.7%)
Eosinophils	Samples	3/45 (6.7%)	3/43 (7%)	0/2 (0%)	2/20 (10%)	0/11 (0%)	1/12 (8.3%)
Basophils	Samples	2/45 (4.4%)	2/43 (4.7%)	0/2 (0%)	1/20 (5%)	1/11 (9.1%)	0/12 (0%)
Plasma cells	Samples	2/45 (4.4%)	2/43 (4.7%)	0/2 (0%)	1/20 (5%)	0/11 (0%)	1/12 (8.3%)
Lymphoid cells	Samples	3/45 (6.7%)	3/43 (7%)	0/2 (0%)	2/20 (10%)	1/11 (9.1%)	0/12 (0%)
Macrophages	Samples	3/45 (6.7%)	3/43 (7%)	0/2 (0%)	1/20 (5%)	2/11 (18.2%)	0/12 (0%)
No pleocytosis	Samples	47/103 (45.6%)	38/91 (41.8%)	8/11 (72.7%)	5/29 (17.2%)	21/26 (80.8%)	12/36 (33.3%)

**Fig. 2:**
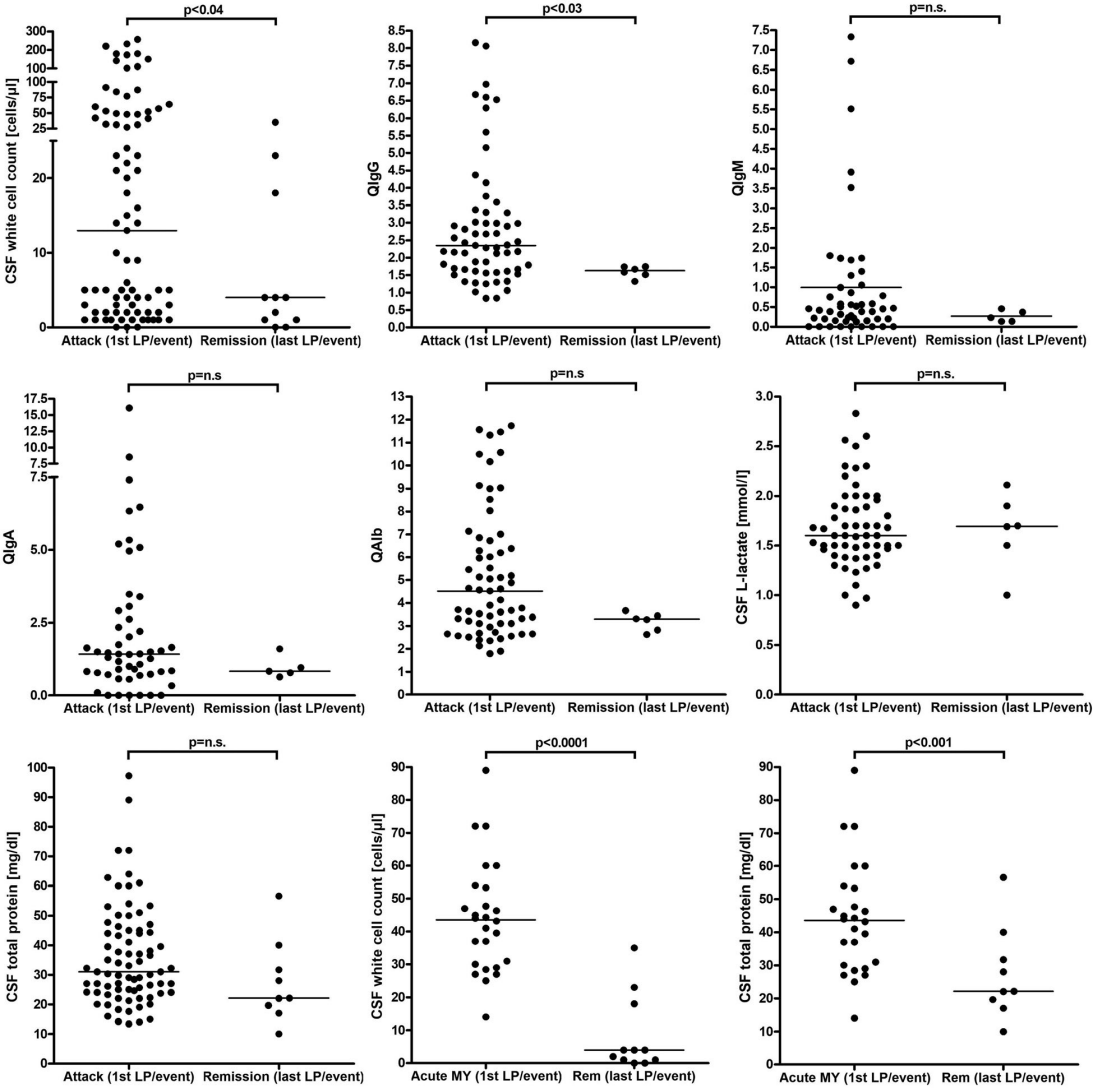
CSF white cell counts, IgG, IgA, IgM and albumin CSF/serum ratios, CSF total protein concentrations, and CSF L-lactate concentrations during acute attacks and remission in MOG-IgG-positive EM. *IgG*/*A*/*M* immunoglobulin G/A/M, *MY* myelitis, *QIgG*/*A*/*M* CSF/serum IgG/A/M ratios, *QAlb* CSF/serum albumin ratio

As in the adult cohort, CSF WCC were significantly higher during acute attacks than during remission (*p* < 0.03) (Fig. 2, Table 1, and Supplementary Figure 1). Activated lymphocytes, plasma cells, macrophages, eosinophils, and basophils were noted only during acute attacks.

**Fig. 3:**
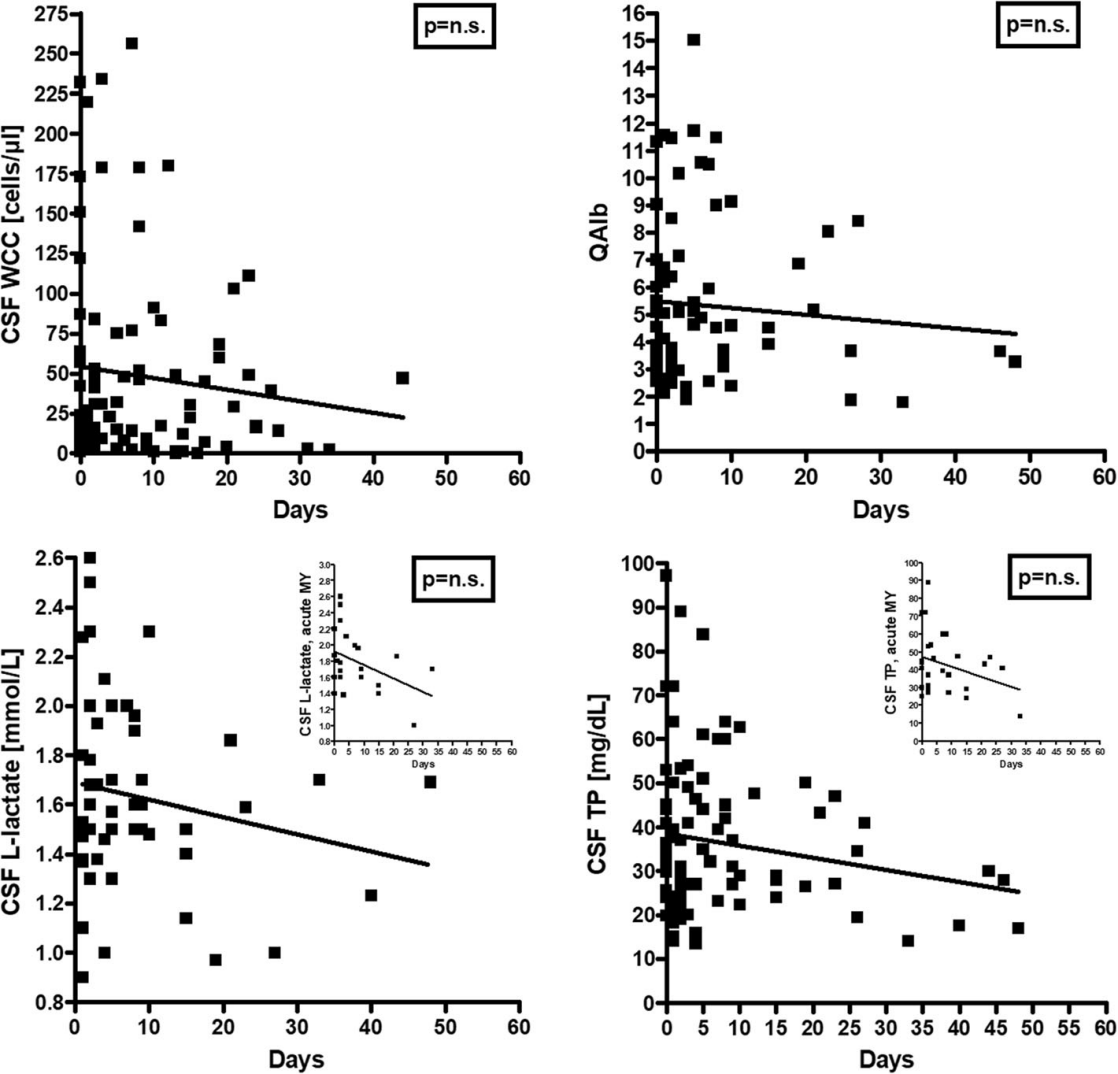
Correlation analyses for CSF white cell counts, QAlb and CSF total protein, respectively, and days since attack onset in patients with acute disease. Although the correlations were not statistically significant, a clear trend towards normal values over time is discernible. Given that clear trend and the significant correlations seen in adults, it is likely that the lack of statistical significance is an effect of the lower number of samples in the pediatric cohort. *QAlb* albumin CSF/serum ratio, *TP* total protein, *WCC* white cell count

**Fig. 4:**
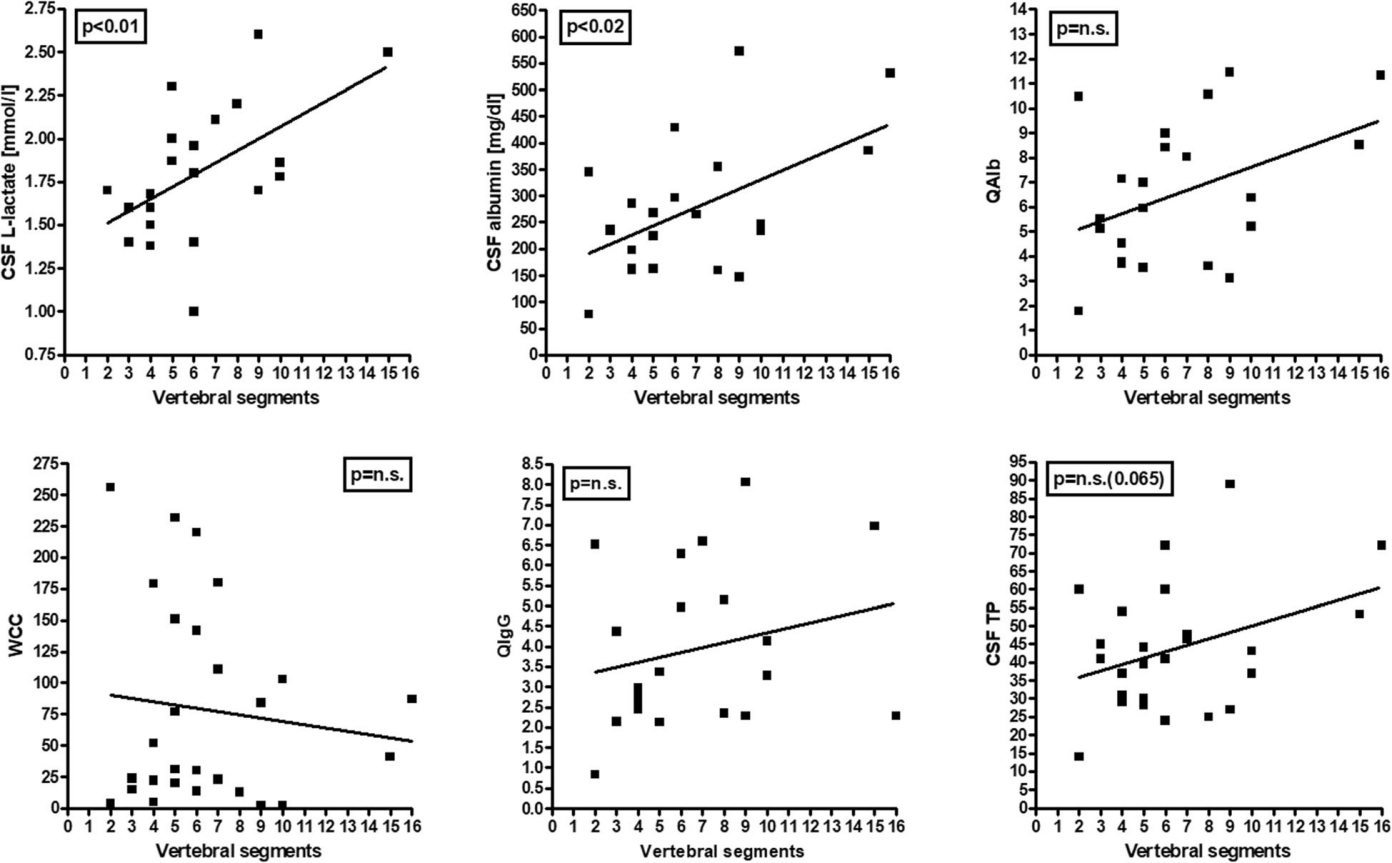
Significant correlation of CSF l-lactate (*r* = 0.549, *p* < 0.01) and CSF albumin concentrations with the spinal cord lesion load (as measured in vertebral segments) in patients with acute MOG-IgG-positive myelitis. *QAlb* albumin CSF/serum ratio, *QIgG* IgG CSF/serum ratio, *TP* total protein, *WCC* white cell count

CSF WCC tended to decline over time after acute attacks (Fig. 3). Different from the adult cohort, no correlation between WCC and the cumulative spinal cord lesion load during acute myelitis attacks was found (Fig. 4). Median CSF WCC during acute attacks were slightly lower in the pediatric cohort than in the adult cohort (81 vs. 100 cells/μl if the first LP per event is considered); however, the difference was not statistically significant.

### Intrathecal IgG synthesis

CSF-restricted OCB were positive in only 11/96 (11.5%) samples (OCB pattern 2 [OCBs that are exclusively present in the CSF but not in serum] in 7/96 or 7.3%; pattern 3 [same as pattern 2 plus identical OCBs in the CSF and serum] in 4/96 or 4.2%), and QIgG was elevated in only 14/78 (18%) (median QIgG 2.4; range 1.5–7) (Table 2). This compares to an almost identical rate of OCB-positive and QIgG-positive samples of 13.2% and 8%, respectively, in the adult cohort [26]. In 7/96 (7.3%) samples, identical OCB in serum and CSF but no CSF-restricted bands were present (pattern 4). Pattern 5, indicating monoclonal gammopathy, was present in 1/96 samples (1%). Overall, 10/72 (13.9%) patients (vs. 16% in adults) showed CSF-restricted OCB at least once, and QIgG was elevated in 13/67 (19.4%) patients (vs. 10.2% in adults; *p* = n.s.) at least once.

**Table 2:** Frequency of intrathecal IgG synthesis, oligoclonal IgG pattern, IgG CSF/serum ratios, intrathecal IgG fractions, absolute amount of locally produced IgG, and absolute IgG concentrations in the CSF and serum

	Units	Total	Attack	Remission	Acute MY subgroup	Acute ON subgroup	Acute BRAIN subgroup
Intrathecal IgG synthesis							
OCB positive or IgG-IF ≥ 10%	Samples	14/97 (14.4%)	14/87 (16.1%)	0/8 (0%)	8/28 (28.6%)	0/27 (0%)	6/32 (18.8%)
OCB positive	Samples	11/96 (11.5%)	11/86 (12.8%)	0/8 (0%)	7/28 (25%)	0/26 (0%)	4/31 (12.9%)
OCB pattern 1	Samples	77/96 (80.2%)	68/86 (79.1%)	7/8 (87.5%)	19/28 (67.9%)	24/26 (92.3%)	24/31 (77.4%)
OCB pattern 2	Samples	7/96 (7.3%)	7/86 (8.1%)	0/8 (0%)	6/28 (21.4%)	0/26 (0%)	1/31 (3.2%)
OCB pattern 3	Samples	4/96 (4.2%)	4/86 (4.7%)	0/8 (0%)	1/28 (3.6%)	0/26 (0%)	3/31 (9.7%)
OCB pattern 4	Samples	7/96 (7.3%)	6/86 (7%)	1/8 (12.5%)	2/28 (7.1%)	2/26 (7.7%)	2/31 (6.5%)
OCB pattern 5	Samples	1/96 (1%)	1/86 (1.2%)	0/8 (0%)	0/28 (0%)	0/26 (0%)	1/31 (3.2%)
OCB pattern 2 or 3	Samples	11/96 (11.5%)	11/86 (12.8%)	0/8 (0%)	7/28 (25%)	0/26 (0%)	4/31 (12.9%)
OCB pattern 3 or 4	Samples	11/96 (11.5%)	10/86 (11.6%)	1/8 (12.5%)	3/28 (10.7%)	2/26 (7.7%)	5/31 (16.1%)
OCB pattern 1, 4, or 5	Samples	85/96 (88.5%)	75/86 (87.2%)	8/8 (100%)	2/28 (75%)	2/26 (100%)	3/31 (87.1%)
QIgG > Q_lim_(IgG)	Samples	14/78 (18%)	13/69 (19%)	0/7 (0%)	8/23 (34.8%)	1/21 (4.8%)	4/25 (16%)
QIgG, all LPs	–	2.3 (0.8–8.2; 76)	2.4 (0.8–8.2; 68)	1.6 (1.3–1.7; 6)	3.13 (0.83–8.06; 22)	1.61 (0.84–8.16; 20)	2.3 (0.95–7.94; 26)
QIgG, if positive	–	2.4 (1.5–7; 14)	2.4 (1.5–7; 13)	n.a. (n.a.; 0)	2.64 (1.79–6.97; 8)	1.53 (1.53–1.53; 1)	2.3 (1.66–4.22; 4)
IgG IF, all LPs	%IgG_CSF_	0 (0-27; 75)	0 (0-27; 67)	0 (0-0; 6)	0 (0-13.2; 22)	0 (0-4.4; 20)	0 (0-27; 25)
IgG IF, QIgG pos	%IgG_CSF_	7.6 (0.03–27; 14)	7.8 (0–27; 13)	n.a. (n.a.; 0)	7.6 (0–13.2; 8)	4.4 (4.4–4.4; 1)	17.9 (4–27; 4)
IgG IF, > 10%	Samples	6/75 (8%)	6/67 (9%)	0/6 (0%)	3/22 (13.6%)	0/20 (0%)	3/25 (12%)
IgG Loc, all LPs	mg/l	0 (0–15.4; 75)	0 (0–15.4; 67)	0 (0–0; 6)	0 (0–4.8; 22)	0 (0–15.4; 25)	0 (0–15.4; 67)
IgG Loc, QIgG pos	mg/l	2.4 (0–15.4; 14)	2.5 (0–15.4; 13)	n.a. (n.a.; 0)	2.7 (0–4.8; 8)	0.6 (0.6–0.6; 1)	3.8 (0.9–15.4; 4)
IgG CSF, all LPs	mg/l	21.6 (5.4–107; 79)	24.6 (5.4–107; 71)	14.8 (10–21.6; 6)	37.6 (5.7–107; 22)	15.1 (5.4–72.6; 20)	22.8 (9–79.9; 29)
IgG CSF, QIgG pos	mg/l	22.5 (13.7–74.3; 14)	22.8 (13.7–74.3; 13)	n.a. (n.a.; 0)	27.9 (20–74.3; 8)	13.7 (13.7–13.7; 1)	22.5 (19–57; 4)
IgG serum, all LPs	g/l	9.6 (3.1–46.9; 78)	9.9 (3.1–46.9; 70)	9 (6.3–13.9; 6)	10.66 (5.27–46.9; 23)	9.29 (5.69–11.6; 20)	9.61 (3.11–15.3; 27)
IgG serum, QIgG pos	g/l	10.4 (6.2–13.8; 14)	10.7 (6.2–13.8; 13)	n.a. (n.a.; 0)	10.7 (6.2–12.7; 8)	9 (9–9; 1)	11.9 (7.8–13.8; 4)
Link index, all	Samples	10/75 (13%)	10/67 (15%)	0/6 (0%)	5/22 (22.7%)	1/20 (5%)	4/25 (16%)
Link index, if positive	Index	0.8 (0.7–0.9; 10)	0.8 (0.7–0.9; 10)	n.a. (n.a.; 0)	0.8 (0.7–0.8; 5)	0.7 (0.7–0.7; 1)	#Value!

Quotients, indices, concentrations, and fractions are given as median and range. *QIgG*/*A*/*M* CSF/serum IgG/A/M ratio, *IgG*/*A*/*M IF* intrathecally produced IgG/IgA/IgM fraction, *IgG*/*A*/*M loc* locally (intrathecally) produced IgG/A/M, *LP* lumbar puncture, *pos* positives

Not only the frequency but also the degree of intrathecal synthesis (IS) was low: QIgG was elevated in only 50% of the OCB-positive samples (which is similar to the 41.2% of samples in the adult cohort). In those samples with elevated QIgG, the intrathecal IgG fraction exceeded the first decile (values < 10% may result from imprecision of nephelometric IgG testing and should thus not be taken as proof of IS according to current guidelines [38]); in only 30% of cases, the median IF-IgG was only 7.6% (range 0.03–27%), and the median absolute amount of intrathecally produced IgG was just 2.4 mg/l (range 0.01 to 15.4 mg/l); similar values were found in the adult cohort (19%, 10.5%, and 2.3 mg/l, respectively) [26].

OCB were detected only in samples obtained during acute attacks (11/86; 12.8%) but not during remission (0/8; 0%; *p* = n.s.). Similarly, larger amounts of intrathecally produced IgG as indicated by an IgG-IF > 10% were found exclusively in samples obtained during acute attacks, just as in the adult cohort (Table 2) [26]. To evaluate whether the frequency of OCB increases with disease duration, we compared samples obtained within the first month since onset and samples obtained more than 1 year after onset but, as in the adult cohort, found no statistically significant difference regarding the rate of OCB-positive samples (13.4% or 9/67, vs. 14.3% or 2/14).

CSF-restricted OCB (pattern 2 or 3) were more frequently seen in the acute MY subgroup than in the acute BRAIN and were completely absent in the acute ON subgroup (25% or 7/28 vs. 12.9% or 4/31 vs. 0% or 0/26; *p* < 0.03) (Table 2 and Fig. 1). Similarly, QIgG was more frequently elevated (34.8% vs. 4.8%; *p* < 0.03) and median IgG CSF/serum ratios (3.13 vs. 1.61; *p* < 0.002) as well as CSF IgG levels (37.6 vs. 15.1 mg/dl; *p* < 0.00008) were significantly higher in the acute MY subgroup than in the acute ON subgroup (Table 2 and Fig. 1). This is similar to what was observed in the adult cohort [26]. However, different from adults, no significant correlation between QIgG and the spinal cord lesion load (as measured in VS) was found (Fig. 4), possibly owing to the lower number of samples in the pediatric cohort.

Median IgG serum concentrations did not differ significantly between acute samples and samples obtained during remission (Table 2) and also not between the acute MY, the acute BRAIN, and the acute ON subgroup (Supplementary Figure 2).

### Intrathecal IgM synthesis

QIgM was increased in 16/65 (25%) samples (median 1.7; range 0.4–7.3) from 16 patients. In those samples with elevated QIgM, the fraction of intrathecally produced IgM varied between 0.2 and 71.3% (median 26.3%), corresponding to a median absolute amount of intrathecally produced IgM of 0.53 mg/l (range 0–4.47), and was > 10% in 13/16 (81%) samples. More samples in the acute MY subgroup than in the acute ON subgroup showed IgM IS (50% vs. 24%), but the difference was not statistically significant. Median QIgM and the median CSF IgM concentration were significantly higher in the MY subgroup than in the ON subgroup (*p* < 0.02 and *p* < 0.006, respectively) (Fig. 1). This might explain the lower frequency of QIgM (12%) and the lower median IgM levels (0.32 mg/l) in the adult cohort, which included a higher proportion of samples from patients with ON than the pediatric cohort.

As with IgG, an intrathecal IgM fraction of > 10% was observed only during acute attacks but not during remission (Table 3). However, based on comparison of the first LP taken during acute attacks (to avoid a bias due to multiple sampling during the same event) and the last LP taken during remission following the same event, the difference did reach statistical significance neither in the pediatric cohort alone nor when data from the pediatric and the adult cohort [26] were pooled. Accordingly, QIgM was also only elevated during acute attacks. Just as in the adult cohort, most LPs did not show any evidence for intrathecal IgM synthesis during acute attacks (QIgM normal and IgM-IF ≤ 10% in 72% and 76.4%, respectively, of acute pediatric samples and 85% and 92.8%, respectively, of acute adults samples), which rather argues against an essential pathogenetic role of intrathecal IgM synthesis.

**Table 3:** Frequency of intrathecal IgM and IgA synthesis, IgM and IgA CSF/serum ratios, intrathecal IgM and IgA fractions, amount of locally produced IgM and IgA, and absolute IgM and IgA concentrations in the CSF and serum

	Units	Total	Attack	Remission	Acute MY subgroup	Acute ON subgroup	Acute BRAIN subgroup
Intrathecal IgM synthesis							
QIgM > Q_lim_(IgM)	Samples	16/65 (25%)	16/57 (28%)	0/6 (0%)	9/18 (50%)	4/17 (24%)	3/22 (14%)
QIgM, all LPs	–	0.4 (0–7.3; 63)	0.5 (0–7.3; 56)	0.2 (0.1–0.5; 5)	0.79 (0–6.72; 17)	0.35 (0–7.33; 16)	0.38 (0–4.17; 23)
QIgM, if positive	–	1.7 (0.4–7.3; 16)	1.7 (0.4–7.3; 16)	n.a.	1.8 (0.45–6.72; 9)	0.76 (0.39–7.33; 4)	1.[74 (1.73–1.81; 3)
IgM IF, all LPs	%IgM_CSF_	0 (0–71.3; 62)	0 (0–71.3; 55)	0 (0–0;5)	0.2 (0–71.3; 17)	0 (0–51.3; 16)	0 (0–53.6; 22)
IgM IF, QIgM pos	%IgM_CSF_	26.3 (0.2–71.3; 16)	26.3 (0.2–71.3; 16)	n.a. (n.a.;0)	25.8 (0.2–71.3; 9)	17.5 (2.3–51.3; 4)	51 (26.8–53.6; 3)
IgM IF, > 10%	Samples	13/62 (21%)	13/55 (23.6%)	0/5 (0%)	7/17 (41.2%)	3/16 (18.8%)	3/22 (13.6%)
IgM Loc, all LPs	mg/l	0 (0–4.5;62)	0 (0–4.5; 55)	0 (0–0; 5)	0 (0–4.5; 17)	0 (0–3.5; 16)	0 (0–2.3; 22)
IgM Loc, QIgM pos	mg/l	0.53 (0–4.47; 16)	0.53 (0–4.47; 16)	n.a.	0.6 (0–4.5; 9)	0.1 (0–3.5; 4)	1.3 (0.2–2.3; 3)
IgM CSF	mg/l	0.43 (0–9.2; 67)	0.5 (0–9.2; 60)	0.41 (0.21–0.6; 5)	0.8 (0–9.2; 17)	0.3 (0–6.89; 16)	0.6 (0–4.2; 27)
IgM serum	g/l	1.04 (0.35–2.6; 69)	1.03 (0.35–2.53; 62)	1.63 (0.9–2.6; 5)	1.09 (0.4–2.53; 20)	0.83 (0.52–1.34; 17)	1.18 (0.35–2.32; 25)
Intrathecal IgA synthesis							
QIgA > Q_lim_(IgA)	Samples	18/65 (28%)	17/58 (29%)	1/5 (20%)	8/18 (44.4%)	2/17 (11.8%)	7/23 (30.4%)
QIgA, all LPs	–	1.4 (0–16.1; 64)	1.4 (0–16.1; 57)	0.8 (0.6–1.6; 5)	2.62 (0–7.4; 17)	0.83 (0–8.52; 16)	1.39 (0–16.06; 24)
QIgA, if positive	–	4.2 (1.3–16.1; 18)	5 (1.3–16.1; 17)	1.6 (1.6–1.6; 1)	5.08 (1.26–7.4; 8)	7.45 (6.38–8.52; 2)	3.06 (1.41–16.06; 7)
IgA IF, all LPs	%IgA_CSF_	0 (0–82.4; 63)	0 (0–82.4; 56)	0 (0–3.4; 5)	0 (0–48.9; 17)	0 (0–63.9; 16)	0 (0–82.4; 23)
IgA IF, QIgA pos	%IgA_CSF_	17.2 (1.1–82.4; 18)	19.5 (1.1–82.4; 17)	3.4 (3.4–3.4; 1)	13.7 (1.6–48.9; 8)	43.6 (23.3–63.9; 2)	36 (1.1–82.4; 7)
IgA IF, > 10%	Samples	13/63 (20.6%)	13/56 (23.2%)	0/5 (0%)	6/17 (35.3%)	2/16 (12.5%)	5/23 (21.7%)
IgA Loc, all LPs	mg/l	0 (0–6.5; 63)	0 (0–6.5; 56)	0 (0–0.1; 5)	0 (0–2.8; 17)	0 (0–3.3; 16)	0 (0–6.5; 23)
IgA Loc, QIgA pos	mg/l	0.8 (0–6.5; 18)	1 (0–6.5; 17)	0.1 (0.1–0.1; 1)	0.8 (0.1–2.8; 8)	2.5 (1.7–3.3; 2)	0.6 (0–6.5; 7)
IgA CSF	mg/l	1.6 (0–17.4; 66)	1.8 (0–17.4; 59)	0.98 (0.9–2.1; 5)	2.95 (0–17.4; 17)	0.9 (0–7.24; 16)	2.12 (0–10.4; 26)
IgA serum	g/l	1.16 (0.05–6.6; 69)	1.16 (0.05–6.6; 62)	1.18 (0.94–2.7; 5)	1.22 (0.69–2.69; 20)	0.84 (0.3–2.61; 16)	1.21 (0.05–6.6; 26)

Quotients, concentrations and fractions are given as median and range. *QIgG*/*A*/*M* CSF/serum IgG/A/M ratio, *IgG*/*A*/*M IF* intrathecally produced IgG/IgA/IgM fraction, *IgG*/*A*/*M loc* locally (intrathecally) produced IgG/A/M, *LP* lumbar puncture, *pos* positives

By contrast, median IgM serum concentrations were significantly lower during acute attacks (*p* < 0.03) (Table 3). However, the latter finding could simply be an effect of the higher rate of female patients (80% vs. 48.4%) and the median age at the time of LP (10 vs. 6 years) in the remission group (normal serum IgM values are higher in female children and tend to rise with age [41, 42]) and/or of the low number of samples in the remission group (*N* = 5) and thus be artificial rather than reflect consumption of IgM during acute attacks. No difference in median IgM serum concentrations was observed in the larger adult cohort [26].

### Intrathecal IgA synthesis

QIgA was increased in 18/65 (28%) samples (median QIgG 4.2; range 1.3–16.1) from 18 patients. Among patients with elevated QIgA, the fraction of intrathecally produced IgA varied between 1.1 and 82.4% (median 17.2), corresponding to an absolute amount of intrathecally produced IgA between 0 and 6.5 mg/l (median 0.8).

QIgA tended to be more frequently elevated in the acute MY subgroup than in the acute ON subgroup (44.4% vs. 11.8%; *p* = n.s.) (Table 3), and median CSF IgA concentrations and IgA CSF/serum ratios were significantly higher (*p* < 0.006 and *p* < 0.03, respectively) in the acute MY subgroup than in the acute ON subgroup (Table 3 and Fig. 1). This might may partly explain the lower values observed in the adult cohort (QIgA elevated and IgA-IF > 10% in 5% and 5%, respectively) which included more samples from patients with acute ON.

IgA-IF exceeded 10% in 13/63 (20.6%) samples with available data and, as IgG-IF and IgM-IF, was seen only in samples obtained during acute attacks (Table 3).

QIgA was elevated during remission in a single sample; however, IgA-IF was low (3.5%) in this case. Median IgA concentrations in the serum did not differ significantly between acute samples and samples obtained during remission (Table 3 and Fig. 2).

### Immunoglobulin (Ig) class patterns

Only three out of 63 (4.8%) samples and three out of 54 (5.6%) patients tested exhibited a so-called three-class immune response as defined by elevation of QIgG, QIgM, and QIgA (2 × IgM-dominant, 1 × IgA-dominant) (no data in the remainder). Based on a stricter definition (IgG-IF, IgM-IF and IgA-IF all > 10%), only 2/63 (3.2%) samples from two patients showed a three-class reaction. In one of these samples, IgG-IF was just borderline positive (10.18%; cut-off: 10%) and IgG-OCB were negative, raising doubts about the presence of IgG IS and reducing the number of samples with a true three-class reaction to one. A three-class reaction was also extremely rare in the adult cohort (1/107 samples [0.9%] or 1/75 [1.3%] patients; possibly artificial due to plasma exchange) [26].

A two-class reaction defined by either positive QIgG and QIgM, positive QIgM and QIgA, or positive QIgG and QIgA was detected in 12/63 (19%) samples and 12/54 (22.2%) patients tested based on QIg > Q_lim_(Ig) (Table 4). In only one of these 12 samples (i.e., in just one out of 63 [1.6%] samples), a dominant IgG two-class reaction was noted; in six, a dominant IgM reaction; and in five, a dominant IgA response. If the stricter definition based on Ig-IF > 10% is used, the number of samples with a positive two-class reaction drops to 6/63 (9.5%) (3 × IgM-dominant, 3 × IgA-dominant; IgG-dominant in none) from six patients (Table 4), which compares to 1/107 (0.9%) (IgM-dominant) samples in the adult cohort. By contrast, an IgG-dominant two-class response has been reported to occur in 20–40% of cases in MS [43].

**Table 4:** Immunoglobulin class response patterns in MOG-IgG-positive EM

	Units	Total	Attack	Remission	Acute MY subgroup	Acute ON subgroup	Acute BRAIN subgroup
a. Based on QIg > Q_lim_(Ig)							
3-class reaction	Samples	3/63 (4.8%)	3/56 (5.4%)	0/5 (0%)	2/18 (11.1%)	0/16 (0%)	1/22 (4.5%)
2-class reaction	Samples	12/63 (19%)	12/56 (21.4%)	0/5 (0%)	6/18 (33.3%)	2/16 (12.5%)	4/22 (18.2%)
IgG + IgM	Samples	2/63 (3.2%)	2/56 (3.6%)	0/5 (0%)			
IgG + IgA	Samples	4/63 (6.3%)	4/56 (7.1%)	0/5 (0%)			
IgM + IgA	Samples	6/63 (9.5%)	6/56 (10.7%)	0/5 (0%)			
1-class reaction	Samples	13/63 (20.6%)	11/56 (19.6%)	1/5 (20%)	6/18 (33.3%)	3/16 (18.8%)	2/22 (9.1%)
Only IgG	Samples	4/63 (6.3%)	3/56 (5.4%)	0/5 (0%)			
Only IgM	Samples	5/63 (7.9%)	5/56 (8.9%)	0/5 (0%)			
Only IgA	Samples	4/63 (6.3%)	3/56 (5.4%)	1/5 (20%)			
b. Based on Ig-IF > 10%							
3-class reaction^a^	Samples	2/63 (3.2%)	2/56 (3.6%)	0/5 (0%)	1/18 (5.6%)	0/16 (0%)	1/22 (4.5%)
2-class reaction^b^	Samples	6/63 (9.5%)	6/56 (10.7%)	0/5 (0%)	2/18 (11.1%)	1/16 (6.3%)	3/22 (13.6%)
IgG + IgM	Samples	0/63 (0%)	0/56 (0%)	0/5 (0%)			
IgG + IgA	Samples	1/63 (1.6%)	1/56 (1.8%)	0/5 (0%)			
IgM + IgA	Samples	5/63 (7.9%)	5/56 (8.9%)	0/5 (0%)			
1-class reaction	Samples	13/63 (20.6%)	13/56 (23.2%)	0/5 (0%)	9/18 (50%)	3/16 (18.8%)	1/22 (4.5%)
Only IgG	Samples	3/63 (4.8%)	3/56 (5.4%)	0/5 (0%)			
Only IgM	Samples	6/63 (9.5%)	6/56 (10.7%)	0/5 (0%)			
Only IgA	Samples	4/63 (6.3%)	4/56 (7.1%)	0/5 (0%)			

^a^IF in samples with a three-class reaction: IgG-IF 10.18%, IgM-IF 71.35% and IgA-IF 48.89%; IgG-IF 26.97%, IgM-IF 53.58% and IgA-IF 11; respectively

^b^IF in samples with a two-class reaction: IgM-IF 27.03% and IgA-IF 19.52%; IgG-IF 23.91% and IgA-IF 35.97%; IgM-IF 36.11% and IgA-IF 12.59%; IgM-IF 26.83% and IgA-IF 82.42%; IgG-IF 10.18% and IgM-IF 71.35%; IgM-IF 50.99% and IgA-IF 62.57%; IgM-IF 51.32% and IgA-IF 23.29%; IgG-IF 26.97% and IgM-IF 53%; respectively

Intrathecal Ig synthesis was restricted to one immunoglobulin class in 13/63 (20.6%) samples (IgG in four; IgM in five; IgA in four) from 13 patients based on Ig CSF/serum ratios and also in 13/63 (20.6%) samples based on Ig-IF > 10% (Table 4).

In three children (vs. two adults [26]) with intrathecal IgM and/or IgA synthesis but no quantitative evidence of intrathecal IgG synthesis, qualitative evidence for intrathecal IgG synthesis, i.e., CSF-restricted OCB, were detectable.

### MRZ reaction

**Fig. 5:**
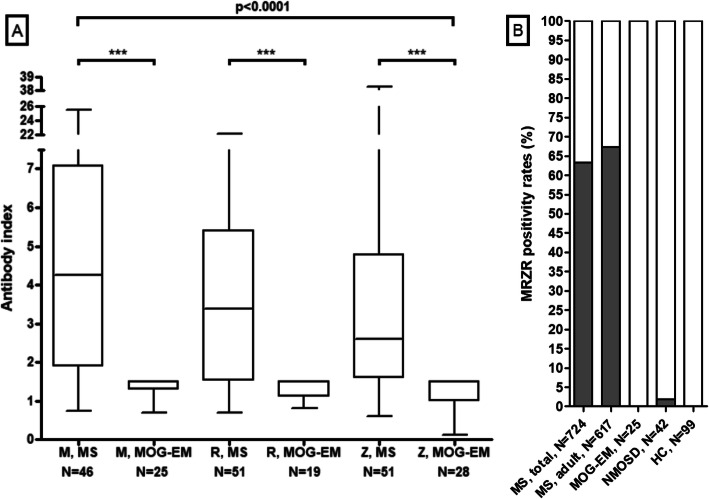
MRZ reaction. Panel **a** shows the antibody indices for M, R and Z in multiple sclerosis (pooled data from ref. [44, 46]) and in samples from MOG-IgG-positive patients (present study). Groups were compared using the Kruskal–Wallis test with Dunn’s post-test. Note that in those cases in which a negative AI was documented but no exact value was available, the AI was set to 1.5, i.e., just below the cut-off for AI positivity (> 1.5); in consequence, the real differences between MOG-EM and MS may be even more pronounced than shown here. Panel **b** shows the frequency of a positive MRZ reaction (MR, MZ, RZ, or MRZ) in MOG-EM (present study), in neuromyelitis optica spectrum disorders (NMOSD), and in healthy controls (HC) (data from [44]). *AI* antibody index, *M* measles virus AI, *R* rubella virus AI, *Z* varicella zoster virus AI

The measles virus (M), rubella virus (R), and varicella zoster virus (Z) reaction (MRZR) was assessed in 29 samples from 25 MOG-IgG-positive patients. All three antibody indices (AI) were tested in 17 samples and two AI in another seven samples; for five LPs, the MRZ reaction (MRZR) was reported as “negative” but the exact AI values not given. A positive MRZ reaction, as defined by the presence of a positive IgG AI for at least two of its three constituents M, R, and Z (i.e., by any of the following combinations: MR, MZ, RZ, or MRZ), is detectable in around 63% of all MS patients [44]. By contrast, the MRZ reaction was absent in all samples in the present cohort of MOG-IgG-positive patients (*p* < 0.000001 when compared to data from a reference paper on the MRZ reaction in MS [45]) (Table 5 and Fig. 5). Similarly, a negative MRZ reaction was found in 62/62 (100%) samples from 48 MOG-IgG-positive patients with available data in the adult cohort.

**Table 5:** MRZ reaction and antibody indices for measles virus (M), rubella virus (R), varicella zoster virus (V), herpes simplex virus (HSV), Epstein Barr virus (EBV), cytomegalovirus (CMV), and *Borrelia burgdorferi* (BB)

	Units	Total cohort
MRZ reaction (M+R, M+Z, R+Z or M+R+Z)	Patients	0/24 (0%)
MRZ reaction (M+R, M+Z, R+Z or M+R+Z)	Samples	0/28 (0%)
AI measles virus (M)	Samples	0/25 (0%)
AI rubella virus (R)	Samples	0/19 (0%)
AI varizella zoster virus (Z)	Samples	0/28 (0%)
Other antibody indices		
AI HSV	Samples	0/21 (0%)
AI EBV	Samples	0/12 (0%)
AI CMV	Samples	0/14 (0%)
AI *B. burgdorferi*, IgG	Samples	0/27 (0%)
AI *B. burgdorferi*, IgM	Samples	1/26 (3.8%)

Intrathecal production of antibodies to measles (with or without concomitant antibodies against rubella and zoster virus) is the most common intrathecal antiviral immune response in MS, both in adults and in children [45]. While it is present in up to 86% of patients with MS, it was present in 0/25 (0%) MOG-IgG samples in the present cohort (*p* < 0.000001 when compared to data from [45]; *N* = 177) and in only 2/61 (3.3%) samples in the adult cohort [26]. A positive rubella virus AI was found in 0/19 (0%) samples (*p* < 0.000001 vs. MS [45]), and a positive varicella zoster virus AI in 0/28 (0%) (*p* < 0.000001 vs. MS [45]).

Median AI for M, R, and Z were significantly lower in patients with MOG-EM than those in two previously published cohorts of patients with MS (*p* < 0.0001) (Fig. 5).

The MRZ reaction was negative not only in OCB-negative samples but also in 3/3 (100%) OCB-positive samples tested for this marker. Similarly, 7/7 (100%) OCB-positive patients tested for MRZ were negative in the adult cohort [26].

### Other antibody indices

A positive IgM AI (AI = 13.28) for *Borrelia burgdorferi* was present in one of 26 samples (3.8%) from 25 patients tested. The *Borrelia* IgG AI was negative in all 27 samples from 27 patents tested, including the *Borrelia* IgM AI-positive sample. None of 14 samples tested exhibited a positive cytomegalovirus (CMV)-IgG AI, 12/12 (100%) samples tested showed a negative IgG AI for Epstein–Barr virus (EBV), and 21/21 (100%) a negative IgG AI for herpes simplex virus (HSV). See also Table 5.

### Blood–CSF barrier integrity

An elevated CSF/serum ratio for albumin, indicating dysfunction of the BCB, was found with 36/79 (45.6%) samples and was present at least once in 32/67 (47.8%) patients tested for this marker (vs. 54.5% in the adult cohort [26]). QAlb ranged between 4.52 and 15.04 (median 6.9) (Table 6). QAlb decreased over time after an attack (Fig. 3) and was normal during remission in all six samples tested. By contrast, BCB dysfunction remained present during remission at almost the same frequency as during acute attacks in the adult cohort (43.5% vs. 46.7%) [26].

**Table 6:** Blood–CSF barrier function, CSF albumin, CSF total protein, and CSF L-lactate in MOG-IgG-positive EM

	Units	Total	Attack	Remission	Acute MY subgroup	Acute ON subgroup	Acute BRAIN subgroup
Blood–CSF barrier function							
QAlb > QAlb(lim)	Samples	36/79 (45.6%)	36/71 (50.7%)	0/6 (0%)	15/23 (65.2%)	6/21 (28.6%)	15/27 (55.6%)
QAlb, all LPs	–	4 (1.8–15; 78)	4.5 (1.8–15; 70)	3.3 (2.6–3.7; 6)	6 (1.8–11.5; 23)	3.2 (1.9–11.6; 20)	4.6 (1.9–15; 27)
QAlb, if positive	–	6.9 (4.52–15.04; 35)	6.9 (4.52–15.04; 35)	0 (0–0; 0)	8 (4.5–11.5; 15)	5.1 (4.6–11.6; 5)	6.7 (4.5–15; 15)
Alb CSF	mg/l	186 (76.8–624; 78)	198 (76.8–624; 70)	131.5 (113–161; 6)	237 (76.8–572; 23)	143 (82–520; 19)	198 (77–624; 28)
Alb serum	g/l	43.35 (30.5–53.9; 78)	43.4 (30.5–53.9; 70)	42.6 (36–45.3; 6)	43.4 (32–53.8; 25)	44 (37.3–48.5; 19)	42.2 (30.5–53.9; 26)
Albumin–cellular dissociation	Samples	8/36 (22.2%)	8/36 (22.2%)	0/0 (0%)	1/15 (6.7%)	4/6 (66.7%)	3/15 (20%)
Combined intrathecal IgG							
synthesis and BCB disruption	Samples	7/36 (19.4%)	7/36 (19.4%)	0/0 (0%)	6/15 (40%)	0/6 (0%)	1/15 (6.7%)
CSF total protein							
CSF TP, all LPs	mg/dl	30.95 (10–97.2; 98)	32.2 (13.4–97.2; 87)	22.1 (10–56.6; 9)	42.1 (14–89; 28)	25.6 (13.4–64; 23)	32.6 (14.2–97.2; 36)
CSF TP, > 100 mg/dl	Samples	0/98 (0%)	0/87 (0%)	0/9 (0%)	0/28 (0%)	0/23 (0%)	0/0 (0%)
CSF TP, elevated (> 45 mg/dl)	Samples	22/98 (22.4%)	21/87 (24.1%)	1/9 (11.1%)	10/27 (37%)	1/25 (4%)	10/35 (28.6%)
CSF TP, if elevated (> 45 mg/dl)	mg/dl	58.3 (46.3–97.2; 22)	60 (46.3–97.2; 21)	56.6 (56.6–56.6; 1)	57 (46.3–89; 10)	64 (64–64; 1)	57 (49–97.2; 10)
CSF TP, elevated (age–adapted^a^)	Samples	59/98 (60.2%)	54/87 (62.1%)	3/9 (33.3%)	55/85 (64.7%)	2/11 (18.2%)	52/77 (67.5%)
CSF TP, if elevated (age-adapted^a^)	mg/dl	41 (26.1–97.2; 59)	42.6 (26.1–97.2; 54)	40 (31.7–56.6; 3)	42 (26.1–97.2; 55)	38.7 (28.4–49; 2)	42.6 (26.1–97.2; 52)
CSF l-lactate							
CSF l-lactate, elevated	samples	22/72 (30.6%)	20/66 (30.3%)	2/6 (33.3%)	10/22 (45.5%)	4/21 (19%)	6/23 (26.1%)
CSF l-lactate, all LPs	mmol/l	1.6 (0.9–2.83; 70)	1.6 (0.9–2.83; 64)	1.7 (1–2.11; 6)	1.74 (1–2.6; 22)	1.5 (0.97–2.56; 19)	1.57 (0.9–2.83 ;23)
CSF lactate, if elevated	mmol/l	2 (1.8–2.83; 22)	2 (1.8–2.83; 20)	2.01 (1.9–2.11; 2)	2.04 (1.38–2.6; 6)	1.53 (1.53–1.53; 1)	1.57 (1.1–2.83; 7)
CSF l-lactate, > 3 mmol/l	samples	0/70 (0%)	0/64 (0%)	0/6 (0%)	0/22 (0%)	0/19 (0%)	0/23 (0%)

Ratios and concentrations are given as median (with range and sample numbers in brackets)

*Alb* albumin, *BCB* blood–CSF barrier, *LP* lumbar puncture, *QAlb* CSF/serum albumin ratio, *TP* total protein

^a^Age-dependent upper reference limits adapted from [47] (0.25 g/l for patients 0.5 months–≤ 6 years at the time of LP, 0.28 g/l > 6–≤ 12 years, 0.34 g/l for > 12–≤ 18 years)

In eight samples (from eight different patients) of 36 tested (22.2%; vs. 34.3% in the adult cohort), an albumin–cellular dissociation (ACD), i.e., compromised integrity of the BCB in the absence of CSF pleocytosis, was found (Table 6).

In line with what was found in the adult cohort, the frequency of BCB dysfunction was higher during acute MY attacks (65.2% [15/23]) than during acute ON attacks (28.6% [6/21]) (*p* < 0.02) (Table 6). As in adults, QAlb was positively linked to the spinal cord lesion load as detected by MRI and measured in VS in patients with acute myelitis (*r*^2^ = 0.175); however, the correlation did not reach statistical significance (*p* = 0.053) (Fig. 4).

### CSF total protein

Total protein (TP) concentrations in the CSF were elevated in 22/98 (22.4%) samples (median 58.3 mg/dl; range 46.3–97.2) and at least once in 19/75 (25.3%) patients with available data. As in the adult cohort, a significant relationship of QAlb and CSF TP levels was found by regression analysis (*r*^2^ = 0.75, *p* < 0.00001) (Supplementary Figure 3). QAlb was elevated in 94.1% of samples with increased CSF TP levels and available data on both parameters. Elevated CSF TP levels were > 45 and < 50 mg/dl (“borderline”) in 4/22 (18.2%) samples, ≥ 50 and ≤ 100 mg/dl in 18/22 (81.8%). Different from the adult cohort, in which 7/48 (14.6%) samples showed CSF TP levels > 100 mg/dl (> 150 mg/dl in 2) [26], CSF TP levels exceed 100 mg/dl in none of the pediatric samples. CSF TP levels were elevated in 21/87 (24.1%) samples obtained during relapse and in a single sample obtained during remission (1/9; 11.1%) (Table 6). Like QAlb, CSF TP levels were more commonly elevated in the acute MY subgroup than in the acute ON subgroup (*p* < 0.006; Table 6); also, median CSF TP levels were higher in the MY subgroup than in the acute ON subgroup (*p* < 0.00008; Fig. 1).

When applying a stricter cut-off of 35 mg/dl as used by some laboratories in children > 12 months of age, the frequency of CSF TP elevation was 38.7% among pediatric samples and 43.8% among adult samples, corresponding to 44.4% of the pediatric patients and 48.4% of the adult patients presenting at least once with elevated CSF TP. If age-partitioned upper reference limits recently proposed by Kahlmann et al. (2017) [47], which were derived from a large European pediatric cohort, were applied (0.25 g/l for patients 6 months–≤ 6 years at the time of LP, 0.28 g/l > 6–≤ 12 years, 0.34 g/l for >12–≤ 18 years; higher upper reference limits have been reported for children < 6 months of age, but no patient was younger than 6 months at the time of LP in the present cohort), the rate of pediatric samples with elevated CSF TP levels was 60.2% and the number of pediatric patients who exhibited elevated CSF TP levels at least once was 64%. The difference between the acute MY and the acute ON group remained highly significant also when applying the 35 mg/dl cut-off or the age-partitioned cut-off intervals proposed by Kahlmann et al. (*p* < 0.0001 and *p* < 0.000006, respectively; Table 6).

Like QAlb, CSF TP levels were negatively yet not statistically significantly correlated with the time (in days) since onset of the last attack, especially in the acute MY subgroup (*r* = − 0.308, *p* = n.s.) (Fig. 3). CSF TP tended to correlate positively with the spinal cord lesions load (*r* = 0.361, *p* = 0.065) (Fig. 4).

### CSF l-lactate

Lactate levels were increased in 22/72 (30.6%; vs. 26.2% in the adult cohort) CSF samples (and at least once in 21/60 [35%] patients tested), with a median concentration of 2 mmol/l (range 1.8–2.83; compared to 2.68 in the adult cohort, *p* < 0.00001) (Table 6 and Fig. 2).

**Fig. 6:**
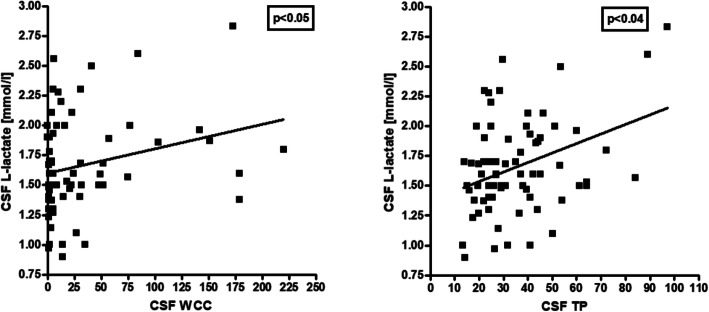
Correlation of CSF l-lactate concentrations with CSF WCC (*r* = 0.257, *p* < 0.04) and CSF TP (*r* = 0.356, *p* < 0.003). *TP* total protein, *WCC* white cell count

As in the adult cohort [26], elevation of lactate levels was more common in the MY subgroup than in the acute ON subgroup (45.5% vs. 19%), although the difference did not reach statistical significance in the smaller pediatric cohort, and CSF lactate concentrations were significantly higher in the MY subgroup (*p* < 0.04) (Fig. 1). Importantly, CSF lactate concentrations were—just as in the adult cohort—significantly correlated with spinal cord lesion load in patients with acute myelitis (*r* = 0.549, *p* < 0.01) (Fig. 4). Similar to the adult cohort, we also found a significant correlation of l-lactate with the CSF WCC (*r* = 0.257, *p* < 0.04) and with CSF total protein (*r* = 0.356, *p* < 0.003) (Fig. 6). CSF l-lactate was elevated in only 16.7% (6/36) samples without pleocytosis but in 44.4% (16/36) of samples with pleocytosis, in 61.5% (8/13) of samples if CSF WCC exceeded 50 cells/μl, and in 71.4% (5/7) if CSF WCC exceeded 100 cells/μl. A similar relationship was also found in the adult cohort (7.8%, 42.9%, 73.9% and 82.4%, respectively). The difference was even more pronounced in the “acute MY” subgroup (0% vs. 58.8%, 75%, and 80%, respectively, in the pediatric and 0% vs. 52.9%, 76.2%, and 86.7%, respectively, in the adult cohort). In patients with pleocytosis, the frequency of samples with elevated CSF l-lactate did not significantly differ between samples with or without neutrophil granulocytes, neither in the total cohort (31.3% [5/16] vs. 44.4% [16/36]) nor in the “acute myelitis” subgroup (33.3% [2/6] vs. 58.8% [10/17]); there was also no significant difference when the pediatric and adult data were pooled (23/59 [39%] vs. 40/92 [43.5%] among all patients and 14/29 [48.3%] vs. 28/51 [54.9%] in the acute MY subgroup). This renders it at least unlikely that granulocytes were the main source of l-lactate in patients with elevated CSF l-lactate levels. When considering not only the presence or absence of neutrophils but absolute neutrophil numbers, a weak trend toward a correlation (*p* = 0.067) was found between CSF l-lactate levels and neutrophil cell numbers in the small subgroup of samples with available data (*N* = 19); however, this was neither seen in the adult cohort nor in a pooled analysis of the pediatric and the adult data. CSF lactate levels showed a trend toward lower values with increasing time (in days) since onset of the last attack (*p* = 0.055) (Fig. 3).

Median CSF l-lactate concentrations during acute attacks were slightly lower in the pediatric cohort than in the adult cohort, if the first LP/event is considered (1.6 vs. 1.815 mmol/l; *p* < 0.02). While CSF l-lactate levels exceed 3 mmol/l in 10/103 (9.7%) patients in the adult cohort [38]; such high levels were noted in none of the pediatric patients.

### First vs. follow-up LP

As in adults [26], the frequency of OCB did not differ significantly between the initial sample and the follow-up samples (8/72 [11.1%] vs. 3/24 [12.5%]). The same holds true if only the first LP performed during each event is considered (11.9% [8/67] vs. 8.3% [1/12]) (Table 7). Similarly, the frequency of IgG-IF elevation, as a quantitative marker for intrathecal IgG synthesis, did not differ significantly between the first LP and follow-up LP as well as the frequencies of pleocytosis, BCB dysfunction, CSF TP elevation, and CSF l-lactate elevation during acute attacks (Table 7).

**Table 7:** CSF findings at the time of the first LP and at follow-up LP. To control for the fact that the number of CSF samples obtained per event differed among patients in the subgroup with follow-up LPs, only the first LP obtained during each attack was taken into account for this analysis

	Units	First LP ever	Follow-up LPs, first LP/event
Pleocytosis, all acute attacks	Samples	41/70 (58.6%)	4/11 (36.4%)
Pleocytosis, acute MY	Samples	21/25 (84%)	1/2 (50%)
Pleocytosis, acute ON	Samples	4/21 (19%)	1/4 (25%)
Pleocytosis, acute BRAIN	Samples	16/24 (66.7%)	2/5 (40%)
OCB, all acute attacks	Samples	8/67 (11.9%)	1/12 (8.3%)
OCB, acute MY	Samples	6/25 (24%)	1/2 (50%)^a^
OCB, acute ON	Samples	0/21 (0%)	0/5 (0%)
OCB, acute BRAIN	Samples	2/21 (9.5%)	0/5 (0%)
IgG-IF > 10%, all acute attacks	Samples	4/53 (7.5%)	6/12 (50%)
IgG-IF > 10%, acute MY	Samples	2/20 (10%)	1/1 (100%)
IgG-IF > 10%, acute ON	Samples	0/18 (0%)	0/1 (0%)
IgG-IF > 10%, acute BRAIN	Samples	2/15 (13.3%)	0/5 (0%)
QAlb > Q_lim_(Alb), all acute attacks	Samples	28/56 (50%)	3/8 (37.5%)
QAlb > Q_lim_(Alb), acute MY	Samples	14/21 (66.7%)	0/1 (0%)
QAlb > Q_lim_(Alb), acute ON	Samples	3/18 (16.7%)	2/2 (100%)
QAlb > Q_lim_(Alb), acute BRAIN	Samples	11/17 (64.7%)	1/5 (20%)
CSF TP elevated, all acute attacks	Samples	16/66 (24.2%)	2/11 (18.2%)
CSF TP elevated, acute MY	Samples	9/23 (39.1%)	1/2 (50%)
CSF TP elevated, acute ON	Samples	1/20 (5%)	0/4 (0%)
CSF TP elevated, acute BRAIN	Samples	6/23 (26.1%)	1/5 (20%)
CSF l-lactate elevated, all acute attacks	Samples	17/49 (34.7%)	2/10 (20%)
CSF l-lactate elevated, acute MY	Samples	9/18 (50%)	1/2 (50%)
CSF l-lactate elevated, acute ON	Samples	2/16 (12.5%)	1/4 (25%)
CSF l-lactate elevated, acute BRAIN	Samples	6/15 (40%)	0/4 (0%)
Time since attack onset, acute LPs	Days	2 (0–33)	4.5 (0–40)

*IgG*-*IF* intrathecal IgG fraction, *OCB* oligoclonal bands, *QAlb* CSF/serum albumin quotient, *TP* total protein, *WCC* white cell count

^a^*p* = n.s.

However, as in the adult cohort, changes were noted in individual patients over time. A total of 20 repeat tests for OCB were performed in 17 patients. OCB turned negative in at least one repeat sample in one patient over the course of the disease (absent 61 days after the initial LP and following treatment with high-dose methylprednisolone) (Supplementary Table 2). In one patient, OCB were negative at first LP and turned positive at repeat examination 24 days later, respectively. Similarly, OCB turned negative in at least one repeat sample in one patient in the adult cohort, and in two adult patients OCB were negative at first LP and turned positive at repeat examination. QIgG was normal in all of the OCB-positive samples, indicating low levels of IgG IS.

In 14 patients, OCB were initially negative and remained negative at follow-up. In two of these patients, OCB pattern changed from pattern 1 to pattern 4, or vice versa, over time (Supplementary Table 2). In one patient, OCB were positive at all (*N* = 2; 2 × pattern 2) LP performed.

Nine of 10 (90%) patients who were tested more than once had a normal IgG CSF/serum ratio both at first LP and at follow-up (as was the case in 24/28 [85.7%] adult patients [26]); in one patient, QIgG remained positive at follow-up. An IgM to IgG IS switch was observed in none of eight patients in whom QIgG and QIgM were both determined more than once (and was rare [1/21] also in the adult cohort).

In MRZ-negative patients, repeat lumbar puncture was reported to increase the sensitivity of MRZ testing in MS due to 'broadening' of the MRZ reaction over time [48]. It is therefore of note that some of the sample used for MRZ testing were obtained at first LP (*N* = 23), whereas others were obtained at follow-up LP (*N* = 6); however, all were negative, irrespective of disease duration at the time of MRZ testing (median 7 days since onset of first attack; range 0–3595), just as in the adult cohort. Moreover, in three patients, MRZ was tested more than once. All four retests (1–2 per patient; median 1.5) in these patients were negative as well (as were 14 follow-up samples from adult patients [26]; the median latency between first and last MRZ testing was 309 days (range 56–724).

### Attack severity

In line with the adult cohort, many CSF parameters assessed were higher and/or more frequently pathologically altered in patients classified as having a severe attack at the time of LP by the treating physician than in patients classified as having mild or moderate disease at the time of LP (Table 8), including, median CSF WCC (28.5 vs. 3 cells/μl; *p* < 0.0000002); median CSF WCC in patients with pleocytosis (48 vs. 12cells/μl; *p* = 0.016); proportion of samples with pleocytosis (73.4% vs. 23.1%; *p* < 0.00001); QIgG values (*p* < 0.004); QAlb positivity rate (60% vs. 30%; *p* = 0.023); median QAlb (5.2 vs. 3.3; *p* = 0.004); and median CSF TP concentration (*p* = 0.008). Moreover, a higher proportion of samples from patients with severe disease at the time of LP exhibited a positive QIgG positivity rate (24% vs. 9%), positive OCB (17% vs. 4%), neutrophils (51.6% vs. 33.3%), and a positive Link index (i.e., IgG index) (20% vs. 5%), although the differences did not reach statistical significance. See Table 8 for details.

**Table 8:** CSF findings and attack severity

	Units	Severe attacks	Mild/moderate attacks	*p* value
WCC, all	Cells/μl	28.5 (0–256; 64)	3 (0–179; 26)	0.0000002
WCC, elevated	Samples	47/64 (73.4%)	6/26 (23.1%)	0.00001
WCC, if elevated	Cells/μl	48 (9–256; 47)	12 (6–179;6)	0.016
Neutrophils, all LPs	Samples	16/31 (51.6%)	4/12 (33.3%)	n.s.
OCB, pattern 2 or 3	Samples	10/58 (17.2%)	1/27 (3.7%)	n.s.
Link index	Samples	9/45 (20%)	1/22 (4.5%)	n.s.
QIgG, all	Ratio	2.7 (0.8–8.1; 47)	1.7 (0.8–8.2;23)	0.004
QIgG, elevated	Samples	11/46 (23.9%)	2/23 (8.7%)	n.s.
QIgG, if elevated	Ratio	2.4 (1.5–7; 11)	2.1 (1.7–2.5; 2)	n.s.
QAlb, all	Ratio	5.2 (1.8–15; 48)	3.3 (1.9–11.6; 23)	0.004
Qalb, elevated	Samples	29/48 (60.4%)	7/23 (30.4%)	0.023
QAlb, if elevated	Ratio	7 (4.5–15; 29)	5.3 (4.6–11.6; 7)	n.s.
CSF TP, all	mg/dl	36.5 (14–97.2; 63)	26.1 (13.4–64; 25)	0.008
CSF TP, elevated	Samples	19/61 (31.1%)	2/25 (8%)	0.027
CSF TP, if elevated	mg/dl	60 (46.3–97.2; 19)	59 (54–64; 2)	n.s.
CSF TP, >100 mg/dl	Samples	0/70 (0%)	0/26 (0%)	n.s.
CSF l-lactate, all	mmol/l	1.7 (0.9–2.8; 44)	1.5 (1–2.6; 22)	n.s.
CSF l-lactate, elevated	Samples	16/44 (36.4%)	4/22 (18.2%)	n.s.
CSF l-lactate, if elevated	mmol/l	2 (1.8–2.8; 16)	2.3 (1.9–2.6; 4)	n.s.
CSF l-lactate, >3 mmol/l	Samples	0/48 (0%)	0/22 (0%)	n.s.

Ratios and concentrations are given as median (with range and sample numbers in brackets)

*IgG*-*IF* intrathecal IgG fraction, *OCB* oligoclonal bands, *QAlb* CSF/serum albumin quotient, *TP* total protein, *WCC* white cell count

### LETM vs. non-longitudinally extensive transverse myelitis

While CSF l-lactate concentrations were significantly correlated (*p* < 0.010) with the spinal cord lesion load and while QAlb (*p* = 0.053) and CSF TP concentrations (*p* = 0.064) tended to correlate with the spinal cord lesion load, no statistically significant differences regarding the frequency of CSF pleocytosis, CSF-restricted OCB, IF-IgG elevation > 10%, BCB dysfunction, or CSF TP elevation were noted when samples were simply stratified into “acute LETM” and “acute NETM” based on the presence or absence of at least one lesion extending over three or more VS (Supplementary Table 3), i.e., if the exact lesion load was not considered. Similarly, no significant difference was found also in the adult cohort (except for CSF l-lactate; *p* < 0.05). However, the rate of samples with pleocytosis, WCC > 100 cells/μl, IgG-IF > 10%, disturbed blood–CSF barrier, or TP elevation, respectively, were all more frequent and CSF WCC and TP concentrations higher in samples obtained during acute LETM when compared to samples obtained during acute NETM, suggesting that the lack of statistical significance may well be an effect of the small sample size in the NETM group.

### Bilateral vs. unilateral ON

As in the adult cohort [26], CSF findings in acute bilateral ON and unilateral ON did not differ significantly, although more samples from patients with bilateral ON exhibited an elevated CSF WCC (Supplementary Table 4).

### Disease course

In line with the adult cohort, no statistically significant differences between samples from patients with a monophasic disease course at last follow-up and patients with a relapsing disease course were observed during acute attacks with regard to the frequency of CSF-restricted OCB, CSF pleocytosis, IgG-IF > 10%, IgM-IF > 10%, IgA-IF > 10%, QAlb elevation, CSF l-lactate elevation, and CSF TP elevation (Table 9).

**Table 9:** CSF findings in patients with monophasic disease and patients with relapsing disease. To control for differences in the number of follow-up samples available per patient, only the first LP performed during each acute event was considered

1st LP/event	Units	Monophasic	Relapsing
Pleocytosis, acute attacks	Samples	26/41 (63.4%)	19/40 (47.5%)^a^
Pleocytosis, acute MY	Samples	14/16 (87.5%)	8/11 (72.7%)
Pleocytosis, acute ON	Samples	3/12 (25%)	2/13 (15.4%)
Pleocytosis, acute BRAIN	Samples	9/13 (69.2%)	9/16 (56.3%)
OCB, acute attacks	Samples	5/39 (12.8%)	4/40 (10%)^a^
OCB, acute MY	Samples	4/17 (23.5%)	3/10 (30%)
OCB, acute ON	Samples	0/12 (0%)	0/14 (0%)
OCB, acute BRAIN	Samples	1/10 (10%)	1/16 (6.3%)
IgG-IF > 10%, acute attacks	Samples	3/36 (8.3%)	2/24 (8.3%)^a^
IgG-IF > 10%, acute MY	Samples	2/16 (12.5%)	1/5 (20%)
IgG-IF > 10%, acute ON	Samples	0/12 (0%)	0/7 (0%)
IgG-IF > 10%, acute BRAIN	Samples	1/8 (12.5%)	1/12 (8.3%)
QAlb > Q_lim_(Alb), acute attacks	Samples	18/36 (50%)	13/28 (46.4%)^a^
QAlb > Q_lim_(Alb), acute MY	Samples	11/16 (68.8%)	3/6 (50%)
QAlb > Q_lim_(Alb), acute ON	Samples	3/12 (25%)	2/8 (25%)
QAlb > Q_lim_(Alb), acute BRAIN	Samples	4/8 (50%)	8/14 (57.1%)
CSF TP elevated, acute attacks	Samples	10/39 (25.6%)	8/38 (21.1%)^a^
CSF TP elevated, acute MY	samples	7/15 (46.7%)	3/10 (30%)
CSF TP elevated, acute ON	Samples	1/12 (8.3%)	0/12 (0%)
CSF TP elevated, acute BRAIN	Samples	2/12 (16.7%)	5/16 (31.3%)
CSF l-lactate elevated, acute attacks	Samples	14/33 (42.4%)	5/26 (19.2%)^a^
CSF l-lactate elevated, acute MY	Samples	8/14 (57.1%)	2/6 (33.3%)
CSF l-lactate elevated, acute ON	Samples	2/10 (20%)	1/10 (10%)
CSF l-lactate elevated, acute BRAIN	Samples	4/9 (44.4%)	2/10 (20%)
Time since attack onset, acute LPs	Days	2 (0-33)	2 (0-40)

*IgG*-*IF* intrathecal IgG fraction, *OCB* oligoclonal bands, *QAlb* CSF/serum albumin quotient, *TP* total protein

^a^*p* = n.s.

The slightly yet statistically non-significantly higher values in the monophasic subgroup noticeable in Table 9 may simply reflect differences in subgroup composition (more samples from patients with acute myelitis [38% vs. 25%], less samples from patients with ON [26% vs. 31.8%], and less samples obtained during mild attacks [4% vs. 25.6%] in the monophasic subgroup; by contrast, the two subgroups did not differ with regard to the proportion of samples from patients untreated at the time of LP [88% vs. 88.6%]). When analyzing the data in a stratified manner according to disease manifestations, the differences were much less pronounced (Table 9). Similarly, when analyzing only patients with severe attacks to eliminate the bias introduced by the overrepresentation of samples obtained during mild attacks in the relapsing subgroup, most differences between the two groups became negligibly small (e.g., pleocytosis: 66.7% vs. 60.7%; OCBs: 14.3% vs. 16%; IgG-IF > 10%: 13.8% vs. 10%; positive QAlb: 47.1% vs. 52%; elevated CSF TP: 25% vs. 33.3%).

### Treatment status

OCB were negative in 12/12 samples in the treated subgroup, QIgG in 8/8, the IgG index in 8/8, CSF l-lactate in 7/9, and CSF TP in 10/11, suggesting a possible effect of treatment on CSF findings. However, no statistically significant differences were found between the treated (*N* = 13) and the untreated subgroup (*N* = 92) with regard to OCB frequency, frequency of QIgG positivity, Link index, QAlb, CSF TP, or CSF l-lactate elevation, possibly due to the low number of samples in the former subgroup. Steroids used included methylprednisolone, prednisolone, and “low dose corticosteroids” (*N* = 11); immunosuppressive and immunomodulatory drugs (*N* = 2) used at the time of LP comprised interferon beta (1×) and intravenous immunoglobulins (1×).

### OCB-positive vs. OCB-negative MOG-EM

Interestingly, OCB were exclusively found during acute attacks (11/11), exclusively during attacks of myelitis or encephalitis (7 × myelitis, 4 × encephalitis, 0 × optic neuritis), and exclusively in untreated patients (11/11). When comparing the CSF findings in the OCB-positive subgroup to that in a similar group of OCB-negative patients (acute and untreated, no ON attacks, no mild attacks), no statistically significant differences were found between the two groups in respect of pleocytosis [73% vs. 70%], median CSF WCC [52 vs. 23 cells/μl], WCC > 150 cells/μl (18% vs. 14%), presence of granulocytes [80% vs 83%], BCB dysfunction [50% vs. 66%], CSF l-lactate elevation [44% vs 35%]. There was no significant difference regarding the median time since onset of the last attack between OCB-positive and OCB-negative samples (8 vs. 2 days). Moreover, as in the adult cohort, the age at the first OCB-positive LP did not differ from that of the first OCB-negative LP in patients who never tested positive for OCB (6.5 years vs. 6 years).

### Acute attacks vs. remission

CSF WCC (*p* < 0.03) and median QIgG (*p* < 0.03) were significantly higher in the subgroup of samples obtained during acute attacks if all samples are taken into account (see Tables 1, 2, 3, 4, and 6). In the larger adult cohort, a higher frequency of CSF l-lactate elevation was found in addition to higher CSF WCC and a higher pleocytosis rate during acute attacks.

When considering only the first LP performed during acute events and the latest LP performed during remission of an event (in order to control for differences in the number of follow-up samples available per patient), some differences were more pronounced (Table 10 and Fig. 2). Of note, almost all parameters assessed were higher or more frequently present during acute attacks. However, statistically significant differences were found only for CSF WCC numbers (*p* < 0.04; vs. *p* < 0.0007 in the larger adult cohort), absolute QIgG values (*p* < 0.03), frequency of BCB dysfunction (*p* < 0.04), possibly owing to the low number of samples in the remission group.

**Table 10:** CSF findings during acute attacks (first LP/event) and during remission (last LP/event)

	Units	Attack, all, first LP/event	Remission, all, last LP/event	*p* values
Pleocytosis	Samples	45/81 (55.6%)	3/11 (27.3%)	*p* = n.s.
WCC	Cells/μl	13 (0–256;81)	4 (0–35;11)	*p* < 0.04
WCC > 100/μl	Samples	10/81 (12.3%)	0/11 (0%)	*p* = n.s.
OCB	Samples	9/79 (11.4%)	0/8 (0%)	*p* = n.s.
QIgG > Q_lim_(IgG)	Samples	12/62 (19.4%)	0/7 (0%)	*p* = n.s.
QIgG	Ratio	2.35 (0.83–8.16; 61)	1.63 (1.32–1.74; 6)	*p* < 0.03
IgG–IF > 10%	Samples	5/60 (8.3%)	0/6 (0%)	*p* = n.s.
QIgM > Q_lim_(IgM)	Samples	15/51 (29.4%)	0/6 (0%)	*p* = n.s.
QIgM	Ratio	0.43 (0–7.33; 50)	0.23 (0.13–0.46; 5)	*p* = n.s.
QIgA > Q_lim_(IgA)	Samples	15/52 (28.8%)	1/5 (20%)	*p* = n.s.
QIgA	Ratio	1.42 (0–16.06; 51)	0.83 (0.64–1.6; 5)	*p* = n.s.
QAlb > Q_lim_(Alb)	Samples	31/64 (48.4%)	0/6 (0%)	*p* < 0.04
QAlb	Ratio	4.52 (1.79–11.73; 63)	3.29 (2.63–3.66; 6)	*p* = n.s.
CSF TP elevated	Samples	18/77 (23.4%)	1/9 (11.1%)	*p* = n.s.
CSF TP concentrations	mg/dl	31 (13.4–97.2; 77)	22.1 (10–56.6; 9)	*p* = n.s.
CSF TP > 100 mg/dl	Samples	0/79 (0%)	0/9 (0%)	*p* = n.s.
CSF l-lactate elevated	Samples	19/59 (32.2%)	2/6 (33.3%)	*p* = n.s.
CSF l-lactate concentrations	mg/dl	1.6 (0.9–2.83; 57)	1.7 (1–2.11; 6)	*p* = n.s.
CSF l-lactate > 3 mmol/l	Samples	0/59 (0%)	0/6 (0%)	*p* = n.s.
Time since attack onset	Days	2 (0–40; 84)	48 (48–3595; 11)	

*IgG*-*IF* intrathecal IgG fraction, *OCB* oligoclonal bands, *QAlb* CSF/serum albumin quotient, *TP* total protein

Finally, separate analyses of the acute MY, the acute ON, and the acute BRAIN subgroups revealed more pronounced differences between the acute phase and remission with respect to some parameters than is evident from the unstratified analysis of the total cohort (see Supplementary Table 1, Table 10, and Fig. 2).

### 'Normal' CSF

A clinically relevant number of CSF samples exhibited no pathological changes. If CSF WCC, OCB, QIgG, Link index, QIgM, QIgA, QAlb, CSF TP, and CSF l-lactate are taken into account, 8/108 (7%; vs. 9% in the adult cohort) samples showed exclusively normal values. Of these eight samples, two were taken during remission and six during acute attacks (no data in 0). Two of the 'acute' yet negative samples belonged to the acute ON group, three to the acute BRAIN group, and one to the acute MY group (no data in 0). If only a basic panel consisting of CSF WCC, CSF TP, and CSF l-lactate is considered (reflecting clinical practice in some non-tertiary centers and in emergency room settings), 29/108 (26.9%; vs. 20.9% in the adult cohort) samples would have been classified as 'normal'. Of those, 21/108 (19.4%; vs. 20.9% in the adult cohort) would have been false-negatives (with the full panel serving as gold standard).

### Influence of age

A significant relationship of age at LP with CSF WCC, QIgG, QAlb, CSF TP, and CSF l-lactate, respectively, during acute attacks was neither found in the pediatric cohort nor in the adult cohort (data not shown). However, an analysis of pooled data from the pediatric and the adult cohort showed a significant relationship of age at LP and CST TP (*p* < 0.0001), QAlb (*p* < 0.0005), and CSF l-lactate (*p* < 0.0003) during acute attacks; a less pronounced relationship was seen for QIgG (*p* < 0.04), whereas no significant relationship was found for CSF WCC (Supplementary Figure 4).

### Quotient diagrams (“reibergrams”)

**Fig. 7:**
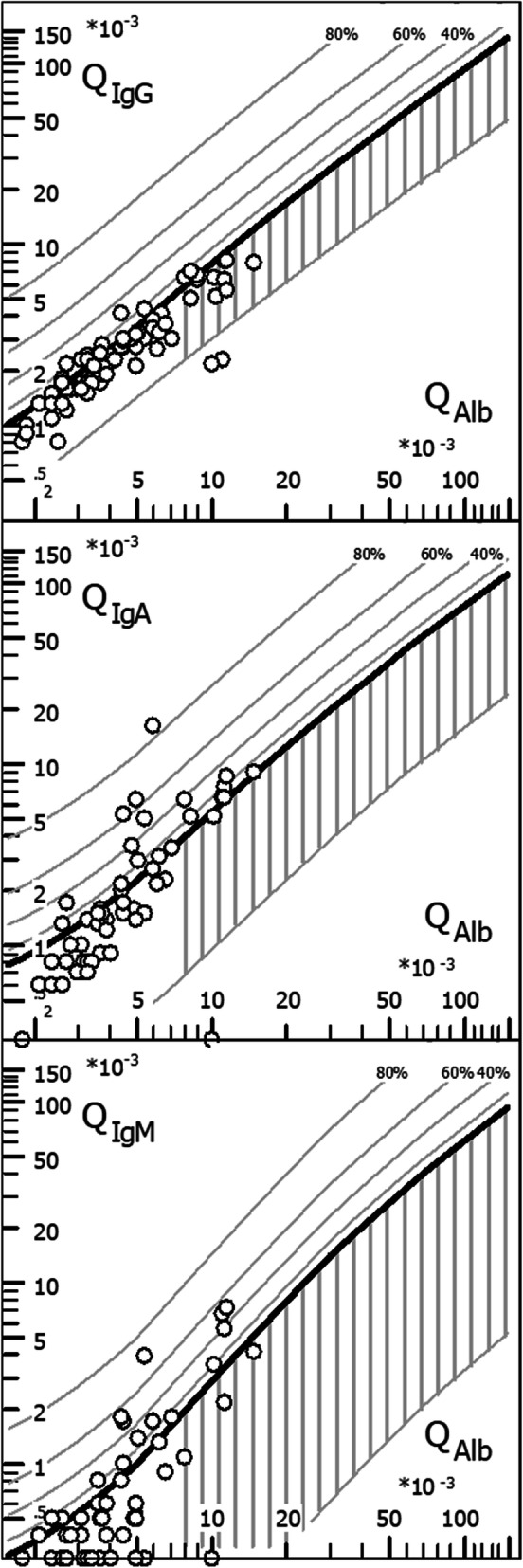
CSF/serum quotient diagrams for IgG, IgM, and IgA (“reibergrams”). Individual CSF/serum ratios of IgG, IgA, and IgM are plotted against CSF/serum albumin ratios. Values above the upper hyperbolic discrimination line, *Q*_lim_, indicate intrathecal synthesis of the respective immunoglobulin (Ig) class. Individual intrathecal fractions, *I*g_IF_, can be directly read by interpolation from the percentiles above Q_lim_ (median values are given in Tables 2 and 3). *IgG*/*A*/*M* immunoglobulin G/A/M, *QIgG*/*A*/*M* CSF/serum IgG/A/M ratios, *QAlb* CSF/serum albumin ratio

Plots of QIgG, QIgA, and QIgM, respectively, against QAlb as a measure of BCB function are shown in Fig. 7.

## Discussion

This study, which consists of two parts, is the largest and most comprehensive study on CSF findings in MOG-EM conducted to date. Considering that children often show clinical and laboratory features different from those in adult patients, that detailed data on CSF findings in MOG-EM in pediatric patients are lacking so-far, and that children are often treated differently, we decided to analyze the pediatric cohort separately. We demonstrate that CSF findings in MOG-EM are clearly different from those reported in MS [35, 49] not only in adults [26] but also in children. Our findings add further evidence in favor of the hypothesis that MOG-IgG-associated EM is a distinct disease entity rather than a subvariant of MS [50–53].

Most strikingly, 85/96 (89%) samples showed no signs of intrathecal synthesis (IS) of IgG, as indicated by a lack of CSF-restricted OCB. This is in stark contrast to MS, in which OCB are detectable in ≥ 95% of cases [35, 49]. Similarly, IS of IgG was absent in 131/151 (87%) samples in the adult cohort. In those samples positive for OCB, the amount of intrathecally produced IgG was often low, as indicated by normal QIgG in 5/10 samples. In the few samples with quantitative evidence of intrathecal IgG synthesis (i.e., with elevated QIgG), the intrathecal IgG fraction was below the second decile in the IgG-specific reibergram in 12/14 (86%) cases and was even below 10% in 8/14 (57%). Moreover, quantitative evidence of intrathecal total IgG synthesis, if present at all, was found only during acute disease attacks.

Of note, OCB were only transiently positive in 2/3 OCB-positive patients tested more than once (Supplementary Table 2). This is in line with findings in the adult cohort but in contrast to the temporal invariance of intrathecal IgG synthesis deemed typical for MS [54], again suggesting a differential immunopathogenesis of the two disorders. Temporal variance of the patients’ OCB status has also been observed in patients with AQP4-IgG-positive NMOSD [17, 55].

The specificity of the intrathecally produced IgG fraction in the few OCB-positive patients with MOG-EM is unknown. MOG-IgG have been previously reported to be present in the CSF in a subset of patients with MOG-EM [2]. However, MOG-IgG is primarily produced in the periphery, as suggested by a negative MOG-specific AI [2]. This corresponds with AQP4-IgG-positive NMOSD, in which the pathogenic antibody is also predominantly produced extrathecally [55–57]. Alternatively, the intrathecally produced IgG could reflect secondary B cell activation, e.g., targeted at antigens unmasked by primary inflammatory tissue damage. Finally, it might be related to coexisting conditions in some patients. Connective tissue disorders (CTD), for example, which relatively frequently co-exist with AQP4-IgG-positive NMOSD [58–60], are associated with OCB in neurological patients in about 25–30% of cases [61, 62]. However, as in the adult cohort, signs of CTD were documented in none of the OCB-positive MOG-EM patients in the present study. OCB can be observed also in CNS infection. While attacks in MOG-EM have been indeed reported to be preceded by viral or bacterial infections (or vaccination) in up to 30% of adult cases [63], no systematic data is available for pediatric patients so far. It is noteworthy in this context that OCB patterns 3 and 4, which indicate systemic immune activation (e.g., during infection) at the time of LP, were present in 11/96 (11.5%) samples.

As in the adult cohort [26], the intrathecal, polyspecific antiviral IgG response typically found in MS (also termed MRZ reaction) [44, 45] was absent in all children tested (*N* = 29), both at onset and at follow-up. Similarly, the MRZ reaction has also been shown to be typically absent in NMOSD (although systematic data on children with NMOSD is missing so far) [45, 46, 64]. The lack of a positive MRZ reaction in patients with MOG-EM, one of the most important differential diagnoses of MS, adds to previous evidence indicating a very high specificity of the MRZ reaction for MS. The MRZ reaction is currently considered the laboratory marker with the highest positive likelihood ratio for MS [44, 46, 64, 65]. Its absence in MOG-IgG-positive patients strongly supports the notion that MS and MOG-EM are two pathophysiologically distinct diseases. If the MRZ results in the pediatric and in the adult cohort [26] are combined, the MRZ reaction was negative in 91/91 (100%) samples, irrespective of OCB status, and in 73/73 (100%) patients tested.

It is a limitation that most studies that evaluated the MRZ reaction in pediatric patients with bona fide MS did not test for MOG-IgG and AQP4-IgG, two markers that have become available only relatively recently [44]. In young children, however, MOG-EM is more frequent than conventional MS, the relative prevalence of which increases with age, and many (and in particular young) children with MOG-EM were falsely diagnosed with MS in the past. In consequence, the rate of MRZR positivity in MS reported in studies published before CBA employing full-length human MOG became available (40–44% [49, 66], compared to around 67% in adults [44]) may well represent an underestimate resulting from accidental inclusion of patients with MOG-EM. By contrast, the prevalence of MOG-EM is many times lower than that of MS in adults. Accordingly, the number of patients with MOG-EM accidentally included in adult studies on MRZR was probably low. This makes the available data on MRZR in adults with MS more reliable.

Due to the retrospective design of this study, we cannot know whether MRZR testing was possibly more frequently considered in patients in whom the differential diagnosis between MOG-EM and MS was particularly difficult. However, as all samples tested were negative for MRZR, the use of the test in such clinically highly relevant subpopulation would only underline its differential diagnostic significance. No other potential biases were identified: MRZ results were provided by 13 different centers (median 1 sample/center; range 1–6), widely ruling out a major center-specific selection bias. MRZ was both negative in samples from the acute MY and BRAIN subgroups (which more frequently exhibited pathological features; 52% of all MRZR-tested samples) and in samples from patients with acute ON (which exhibited overall less pronounced CSF pathology; 48% of all MRZR-tested samples). Median age of the MRZR-tested patients was 8 years (range 1–17), which was very similar to that among all samples and that in the non-tested subgroup (6 years, range 0–17; *p* = n.s.). The MRZR-tested cohort comprised an approximately equal proportion of female and male patients (55.2%% vs. 44.8%%), and the proportion of female patients among all MRZR-tested samples was virtually identical to that among all samples (55.2% vs. 55.6%). This strongly argues against an effect of age and sex. Finally, MRZR was negative both in all OCB-positive and all OCB-negative samples tested and the proportion of OCB-positive samples tested for MRZR (27%) did not differ from the total proportion of samples tested for MRZR (27%). Similarly, MRZR was also negative in all tested OCB-positive samples in the adult cohort.

Finally, the possibility of false-positive results needs to be appreciated when considering why a few patients showed signs of intrathecal IgG synthesis. QIgG results should be interpreted with caution whenever IgG-IF values are below 10%, owing to the limited precision of IgG measurements in serum and CSF, which are inherent to the methods (mostly nephelometry) used, if supporting evidence from OCB determination (which, performed properly, is substantially more sensitive than QIgG) is lacking. Current guidelines on CSF diagnosis set the upper limit for imprecision at 7–10%, for incorrectness at 10%, and for deviation between single measures at 24–30% [38]. In fact, IgG-IF was below 10% and OCB were negative or were not tested in nine samples from nine patients. If QIgG results not supported by either an IF-IgG > 10% or CSF-restricted OCBs are not considered true-positive (as recommended by some authors [38]), QIgG was positive only in 5/78 (6.4%) samples from four out of 67 (6%) patients tested at least once, which is nearly identical to the frequency found in the adult cohort (6%) [26].

A substantial number (*N* = 16, or 25%, based on Q_lim_, and *N* = 13, or 21%, based on IgM-IF > 10%) of pediatric samples showed evidence of possible low intrathecal production of IgM antibodies. Like IgG, IgM synthesis was also found only during acute attacks (just as in the adult cohort [26]). Interestingly, in 5/63 (7.9%) cases, IS exclusively of IgM but not of IgG was observed, whereas in the remaining cases IgM IS was accompanied by IgG IS (*n* = 2) or IgA IS (*n* = 6) or both IgG IS and IgM IS (*n* = 3). By contrast, isolated IgM IS is atypical in MS and should prompt doubt regarding that diagnosis. The specificity of the IgM antibodies in our MOG-EM patients is unknown. While in the adult cohort, five of the 16 CSF samples with elevated QIgM were available for retrospective testing but were all negative for MOG-IgM (testing performed after preabsorption of total IgG to rule out false-positive or false-negative IgM results [67]), no paired CSF/serum samples were retrospectively available in the pediatric cohort. To the best of our knowledge, there are also no reports on marked IgM deposition in MOG-EM lesions (as seen in NMOSD [68]). A previous study on mostly adult patients found MOG-IgM in 2/23 MOG-IgG-positive serum samples but did not test for MOG-IgM in the CSF [3]. Alternatively, blood contamination could have played a role in a subset of cases. QIgM is much more sensitive to blood contamination than QIgG, and a relevant number of erythrocytes were detectable in at least 2/14 QIgM-positive patients (no or only minor contamination in 12). Finally, the intrathecal IgM fraction was < 10% in 3/16 QIgM-positive patients. In patients with such low IF values, false-positive QIgM results (owing to unavoidable imprecision of IgM measurements [38]) cannot be ruled out.

Blood–CSF barrier disturbance as indicated by QAlb elevation was common and more severe than in MS. While QAlb is normal in around 90% of MS patients [35, 40], it was elevated in almost every second MOG-EM sample both in the pediatric and in the adult cohort. This is very similar to the high frequency of BCB disruption seen in a study on mostly adult patients with AQP4-IgG-positive NMOSD (51%) [55]. This is of importance, since extrathecally produced MOG-IgG might gain access to the CNS via regions of disturbed BCB function. In the adult cohort, long-lasting BCB damage in MOG-EM (as previously seen also in AQP4-IgG-positive NMOSD [55]) was observed. It is unclear whether this reflects slow recovery from severe damage or rather ongoing subclinical inflammation. In agreement with the latter notion, MOG-IgG (just like AQP4-IgG [69]) remains detectable, partly at high levels, in many patients with MOG-EM also during remission. Different from the adult cohort, QAlb elevation was found exclusively during acute attacks in the pediatric cohort. However, the pediatric 'remission group' was too small to draw definite conclusions in that regard. More differences between adults and children were observed in terms of BCB dysfunction: Absolute QAlb values were significantly lower in the pediatric cohort than in the adult cohort (median 6.46 vs. 4.52, based on results from the first LP/event; *p* < 0.0001) and QAlb values exceeding 12 × 10^−3^, which were found in more than a quarter of the adult patients with elevated QAlb, were present only in a single sample (2.9%) in our pediatric patients (*p* < 0.002). Nonetheless, brain–CSF barrier dysfunction was not rare. Based on age-related upper reference intervals, QAlb was elevated in almost every second sample.

It should be kept in mind, as a general rule, than QAlb rather than CSF TP levels should be used to evaluate blood–CSF barrier function, since CSF TP levels depend on both intrathecal synthesis and serum levels [38, 43]. However, in both the pediatric and the adult cohort, a close and statistically highly significant relationship between QAlb and CSF TP levels was found, suggesting that CSF TP levels are mainly dependent on brain–CSF barrier function in MOG-EM. Accordingly, QAlb was elevated in nearly all samples with elevated CSF TP.

Age may also influence the extent of CSF pathology. Regression analyses of the pooled dataset from the pediatric and the adult cohort suggest a relationship of age and QAlb, CSF TP, CSF l-lactate and, possibly, QIgG in patients with MOG-EM.

In children, the CSF TP upper reference is age-dependent, with very high values in newborns, a rapid decline over the first months, and, after remaining low for some years, a gradual increase until adult values are reached, though some differences in dynamics observed exist between studies [47, 70]. It has thus been suggested that the standard use of adult reference values in the pediatric population may not be ideal [70]. In consequence, we provide additional data on age-partitioned reference intervals for CSF TP in this study [47]. Application of age-dependent reference limits resulted in a slightly higher frequency of TP elevation in the pediatric cohort despite slightly lower median absolute CSF TP levels (different from the adult cohort, CSF TP levels exceeded 100 mg/dl in none of the children tested). Similarly, age-adapted upper reference limits were reported for QAlb and l-lactate and applied in our study. Age-adapted l-lactate CSF levels were elevated in almost one-third of our pediatric patients (Table 6 and Fig. 2), which was not significantly different from the proportion seen in adults (26%). This is similar to AQP4-IgG-positive NMOSD [55] but in contrast to MS, in which l-lactate levels are usually normal [71]. In line with the lower CSF l-lactate levels reported in healthy children, median CSF l-lactate levels were slightly lower in children with MOG-EM and CSF lactate levels > 3 mmol/l as observed in some of the adult patients were present in none of the children tested. CSF l-lactate levels were significantly higher in patients with acute myelitis than in acute ON in the adult cohort, and a trend toward higher values was observed also in the smaller pediatric cohort. Importantly, CSF l-lactate levels were strongly correlated with the cumulative spinal cord lesion load at the time of acute myelitis in both adult (*p* < 0.0001) and pediatric patients (*p* < 0.01) (Figs. 1 and 4). In contrast to albumin and TP, median l-lactate levels are physiologically higher in the CSF than in the serum and independent from peripheral l-lactate levels [72, 73]. This makes it per se highly unlikely that the observed increase in CSF l-lactate levels was related to the BCB dysfunction observed in many MOG-IgM patients. In fact, no correlation of CSF l-lactate levels with QAlb was found. It thus seems more likely that CSF l-lactate and QAlb independently reflect the extent of intrathecal inflammation. CSF l-lactate levels were also correlated with the CSF WCC both in adults and children.

Granulocytes are a known source of CSF l-lactate [74–78]. However, neither the frequency of l-lactate elevation nor median l-lactate levels differed significantly between samples with and without granulocytes, both in the pediatric and in the adult cohort. Moreover, no statistically significant correlation between CSF granulocyte counts and CSF l-lactate levels could be demonstrated (although a trend was noticeable in the pediatric cohort). As a limitation, the number of samples with exact data on CSF granulocyte numbers was small. CSF l-lactate is thought to be produced also by astrocytes following glutamate stimulation [79, 80]. In NMOSD, in which we could also demonstrate a correlation between CSF l-lactate levels and the spinal cord lesion load [55], AQP4-IgG has been reported to result in increased extracellular glutamate concentrations due to coupled endocytosis of AQP4 and the excitatory amino acid transporter 2 (EAAT2) [81]. However, there is no evidence so far for marked astrocytic dysfunction (e.g., resulting from inflammatory bystander damage) in MOG-EM. As previously discussed [82], an increase in extracellular glutamate could exert potentially detrimental effects also by overstimulating glutamate receptors in neurons and MOG-expressing oligodendrocytes [81]. It also renders oligodendrocytes susceptible to immunoglobulin-independent (alternative pathway) complement attack [81, 83]. Finally, neurons may switch to glycolysis, in particular if their capacity to metabolize anaerobically the lactate of astrocytic origin is exhausted [80]. Further studies are needed to better characterize the sources of intrathecal l-lactate in MOG-EM.

An elevated WCC was found in about 60% of samples from pediatric patients with active disease at the time of LP, which is identical to the rate found in adult patients [38]. Median WCC did also not differ significantly between children and adults. Among CSF white cells, lymphocytes and monocytes were predominant, followed—as in adults—by neutrophils, an immune cell type never observed in MS (but in around 50% of samples from patients with acute attacks of AQP4-IgG-positive NMOSD [55]). In line with our demonstration of a lack of intrathecal MOG-IgG production in MOG-EM in a previous study [2], the lack of OCB and the normal QIgG values in most patients, and the lack of a positive MRZ reaction in our patients, antibody-secreting plasma cells were reported only for 4.4% of all samples, which is almost identical to the rate found in adults (3.9%). The proportion of samples with activated lymphocytes (6.7%) was insignificantly lower than in the adult cohort (15.6%), which exhibited a frequency similar to that in AQP4-IgG-positive NMOSD (20.5%), and was much lower than that usually seen in MS (> 75%) [40].

While a CSF WCC > 50 cells/μl is rare in MS and should prompt physicians to challenge the diagnosis, white cell numbers > 50 were observed in 22% of all pediatric samples, in 28% of all samples with pleocytosis, and in as many as 48% of those taken during acute myelitis (50% if lesions were longitudinally extensive). In the acute MY subgroup, WCC exceed even 100 cells/μl in every third patient; such high white cell numbers are virtually never seen in MS. These numbers are almost identical to those in the adult cohort (19%, 27%, 46%, and 52%, respectively) [26].

Neutrophil granulocytes or elevated l-CSF lactate levels, two laboratory features of bacterial CNS infection, were frequently observed during acute attacks both in children and adults. Granulocytes are also detectable in the CSF during very early-stage viral encephalomyelitis. Given the fact that MOG-EM attacks (in common with NMOSD attacks [17, 63]) are often preceded by infections [3, 4] which may result in fever or blood leukocytosis, this might well lead to the false suspicion of infectious disease in some cases. However, in most samples, both CSF lactate levels and absolute CSF white cell numbers were much lower in MOG-EM than in typical bacterial meningitis. While lactate concentrations exceeded the age-dependent reference range in 31% of samples in the pediatric cohort and 26% in the adult cohort, lactate concentrations > 3 or > 4 mmol/l, as seen in a majority of patients with acute bacterial meningitis, were absent in all samples tested in the pediatric cohort and in over 90% in the adult cohort.

Eosinophilic infiltration is not a typical feature of MOG-EM [84, 85]. In line with that observation, eosinophils were absent in all but three samples in the present pediatric cohort and in all but two samples in the adult cohort. This is similar to MS, in which eosinophils are typically absent in the CSF, too. By contrast, previous studies have demonstrated the presence of eosinophil attractants in the CSF of patients with NMO [86], eosinophilic infiltration in NMO lesions [68], and the presence of eosinophils in 10–15% of acute CSF samples from patients with AQP4-IgG-positive NMOSD [55].

It is of note that CSF pathology was strikingly less severe and less frequent in samples obtained during acute attacks of ON than in acute myelitis (Fig. 1), both in children and adults. Patients presenting with isolated brain lesions exhibited CSF alterations more severe than in ON but mostly less severe than in myelitis. These findings are well in line with the fact that the lesion volume is rather small in ON compared with myelitis (median lesion load six VS; up to 16 VS; LETM in 83%). Moreover, lumbar CSF in general does not reflect supratentorial lesions well due to its remoteness from the actual site of inflammation (so-called caudal–rostral CSF gradient). Moreover, we found highly significant differences in terms of CSF pathology (especially with regard to WCC, pleocytosis rate, QAlb, and TP) between attacks classified as “severe” by the treating physicians and attacks classified as only “mild” or “moderate” in this study. Future studies should attempt to define more objective measures for attack severity classification.

With the re-integration of OCB in the latest revision of the diagnostic criteria for MS [87] and the demonstration of substantial differences in CSF profiles between MS and its most important mimics [44, 46, 55, 64, 88–90], LP may be performed more often in the future. Although LP is a relatively safe procedure and routinely used in many countries, adverse event such as headache (post-puncture CSF pressure syndrome, the frequency of which can be substantially lowered by use of so-called atraumatic 22–24 gauge needles with conical tip and lateral opening [“Sprotte needles”]), radicular symptoms, non-specific back pain, disc prolapse or aseptic disc necrosis (extremely rare), bleeding, or infection rarely occur and a number of absolute (increased intracranial pressure with progressive herniation as indicated clinically and/or by MRI or CT; inflammatory infiltration of the skin in the puncture area) and relative (platelet counts < 50 GPt/L; therapeutic heparinization; oral anticoagulation) contraindications exist [40]. In consequence, patients should be thoroughly examined and contraindications carefully considered before performing LP. For a more detailed review of LP techniques and the prevention and management of complications, see [40, 43].

## Strengths and limitations

We count among the strengths of this study the high number of pediatric patients included (given the rarity of the disease), the large number of both samples and parameters analyzed, and the stratified analysis taking into account the clinical presentation at the time of LP. It is a potential limitation that our study included a large number of centers. However, the rarity of MOG-IgG-associated EM means that monocenter studies cannot be performed if sufficient sample numbers are to be analyzed. Moreover, the multicenter approach reduces the risk of selection bias. Finally, no standardized MRI protocols were used. Although the correlation of lactate and TP levels with the spinal cord lesions lesion load found in our cohort is intriguing, further studies are needed to confirm this finding in a prospective and more standardized fashion.

## Conclusion

In summary, our study, the first to review comprehensively and systematically the CSF findings in children with MOG-EM in a large cohort of patients of mainly Caucasian descent, demonstrates that (i.) in sharp contrast to classic MS, intrathecal IgG synthesis is rare in MOG-IgG-associated EM, as shown both qualitatively and quantitatively; (ii.) if present, intrathecal IgG synthesis is low in most patients, partly transient, and restricted mainly to acute attacks (again in contrast to MS); (iii.) CSF findings in acute myelitis differ substantially and significantly from those in acute ON (normal CSF findings are not rare in ON and do not exclude the diagnosis); (iv.) CSF findings in “monophasic” MOG-EM are not significantly different from those in relapsing MOG-EM; (v.) different from MS, the degree of CSF alteration depends on disease activity and attack severity (and could thus have potential prognostic value); (vi.) in patients with acute myelitis, CSF l-lactate levels as well as CSF albumin and CSF TP levels correlated with the spinal cord lesion load (again suggesting a potential prognostic value of LP in MOG-EM); (vii.) CSF white cell numbers in MOG-EM may well exceed those typically observed in MS, in particular in acute myelitis (> 50 cells/μl in around 50% during acute LETM); (viii.) a lack of pleocytosis, on the other hand, does not rule out the condition but is a frequent finding (around 80% in acute ON); (ix.) the intrathecal, polyclonal antiviral immune response (so-called MRZ reaction) discriminates sharply between MOG-EM and MS; and (x.) neutrophilic pleocytosis and elevated l-lactate CSF concentrations render the condition—just like AQP4-IgG-positive NMOSD—a relevant differential laboratory diagnosis of (especially nonpurulent or chronic) bacterial infection in a subset of patients. In many respects, CSF findings in MOG-EM share much more similarities with NMOSD than with MS. Our data may help to improve the differential diagnosis of MOG-EM and MS and to extend our understanding of the immunopathology of this newly described entity. Except for lower QAlb values and l-lactate levels during acute attacks, CSF findings in the pediatric cohort did not differ substantially from those in the adult cohort. A detailed analysis of the CSF findings in adult patients with MOG-EM can be found in Part 1 of this article series [26].

## Supplementary information

**Additional file 1: Supplementary Figure 1.** CSF white cell counts in the ‘acute MY subgroup’, the ‘acute BRAIN subgroup' and the ‘acute ON subgroup’.

**Additional file 2: Supplementary Figure 2.** No marked differences in serum IgG, IgM, IgA and albumin levels between the ‘acute MY’, the ‘acute ON’ and the ‘acute BRAIN’ (B) subgroup, except for slightly higher median IgM values in the ‘acute BRAIN’ subgroup as compared to the acute ‘ON’ subgroup.

**Additional file 3: Supplementary Figure 3.** Regression analysis of QAlb and CSF total protein, demonstrating a close relationship between the two parameters (r^2^=0.75, *p*<0.00001).

**Additional file 4: Supplementary Figure 4.** Influence of age at LP on CSF parameters as detected by a pooled linear regression analysis of data from the pediatric and the adult cohort (r^2^=0.089 for CSF TP, *p*<0.0001; r^2^=0.064 for QAlb, p=0.0004; r^2^=0.027 for QIgG, p<0.04; r^2^=0.097 for CSF L-lactate, p=0.0002). By contrast, no significant relationship was observed when the two cohort were analysed separately (not shown).

**Additional file 5: Supplementary Table 1.** CSF findings during acute attacks and during remission in the ‘acute MY’ subgroup, the ‘acute ON’ subgroup and the ‘acute BRAIN’ subgroup (stratified results from Table 10).

**Additional file 6: Supplementary Table 2.** Variations in OCB positivity over time.

**Additional file 7: Supplementary Table 3.** CSF findings in MOG-IgG-positive acute longitudinally extensive transverse myelitis (LETM) and MOG-IgG-positive non-longitudinally extensive transverse myelitis (NETM).

**Additional file 8: Supplementary Table 4.** CSF findings in MOG-IgG-positive acute bilateral ON and in MOG-IgG-positive acute unilateral ON.

## Data Availability

The datasets generated and/or analyzed during the current study are not publicly available but can be obtained by qualified researchers from the corresponding author upon reasonable request.

## Abbreviations

ADEM: Acute disseminated EM
AI: Antibody index
AQP4: Aquaporin-4
BCB: Blood–CSF barrier
CBA: Cell-based assay
CSF: Cerebrospinal fluid
CTD: Connective tissue disorders
EM: Encephalomyelitis
IF: Intrathecal fraction
IgG/M/A: Immunoglobulin G/M/A
IHC: Immunohistochemistry
IS: Intrathecal synthesis
LETM: Longitudinally extensive transverse myelitis
LP: Lumbar puncture
MOG: Myelin oligodendrocyte glycoprotein
MOGAD: MOG antibody-associated disease
NETM: Non-extensive transverse myelitis
NMO: Neuromyelitis optica
NMOSD: NMO spectrum disorders
OCB: Oligoclonal bands
ON: Optic neuritis
QAlb: Albumin CSF/serum ratio
QIgG: IgG CSF/serum ratio
QIgM: IgM CSF/serum ratio
QIgA: IgA CSF/serum ratio
rON: Recurrent optic neuritis
TP: Total protein
VS: Vertebral segments
