# Supplementary material for: Cerebrospinal fluid findings in patients with myelin oligodendrocyte glycoprotein (MOG) antibodies. Part 2: Results from 108 lumbar punctures in 80 pediatric patients
Source: J Neuroinflammation. 2020 Sep 3;17:262. doi: 10.1186/s12974-020-01825-1 (PMC7470445; doi:10.1186/s12974-020-01825-1)
Supplement: Supplementary file 1 — Additional file 1: Supplementary Figure 1. CSF white cell counts in the ‘acute MY subgroup’, the ‘acute BRAIN subgroup' and the ‘acute ON subgroup’. [file 12974_2020_1825_MOESM1_ESM.pdf]

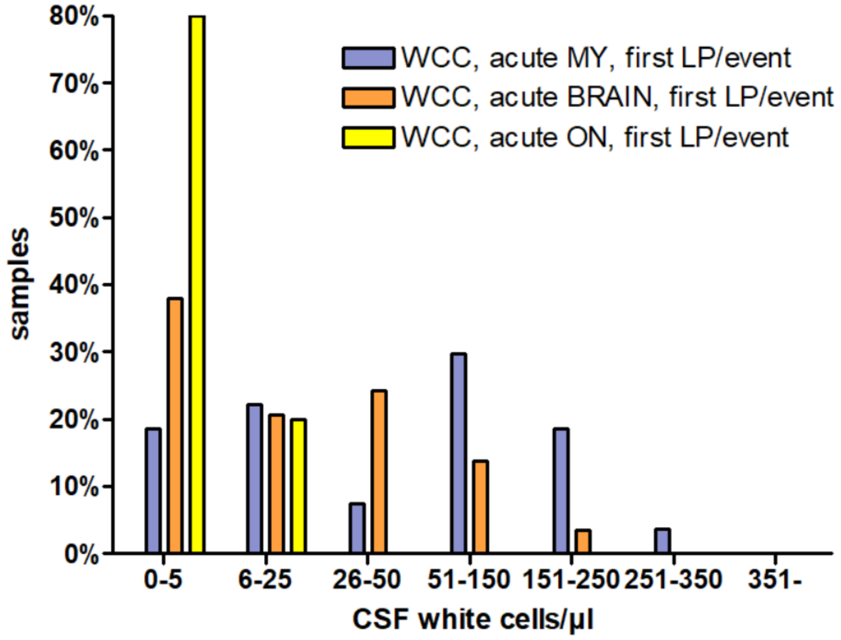

Supplementary Figure 1. CSF white cell counts in the ‘acute MY subgroup’, the ‘acute BRAIN subgroup’, and the ‘acute ON subgroup’.
