# Supplementary material for: Cerebrospinal fluid findings in patients with myelin oligodendrocyte glycoprotein (MOG) antibodies. Part 2: Results from 108 lumbar punctures in 80 pediatric patients
Source: J Neuroinflammation. 2020 Sep 3;17:262. doi: 10.1186/s12974-020-01825-1 (PMC7470445; doi:10.1186/s12974-020-01825-1)
Supplement: Supplementary file 2 — Additional file 2: Supplementary Figure 2. No marked differences in serum IgG, IgM, IgA and albumin levels between the ‘acute MY’, the ‘acute ON’ and the ‘acute BRAIN’ (B) subgroup, except for slightly higher median IgM values in the ‘acute BRAIN’ subgroup as compared to the acute ‘ON’ subgroup. [file 12974_2020_1825_MOESM2_ESM.pdf]

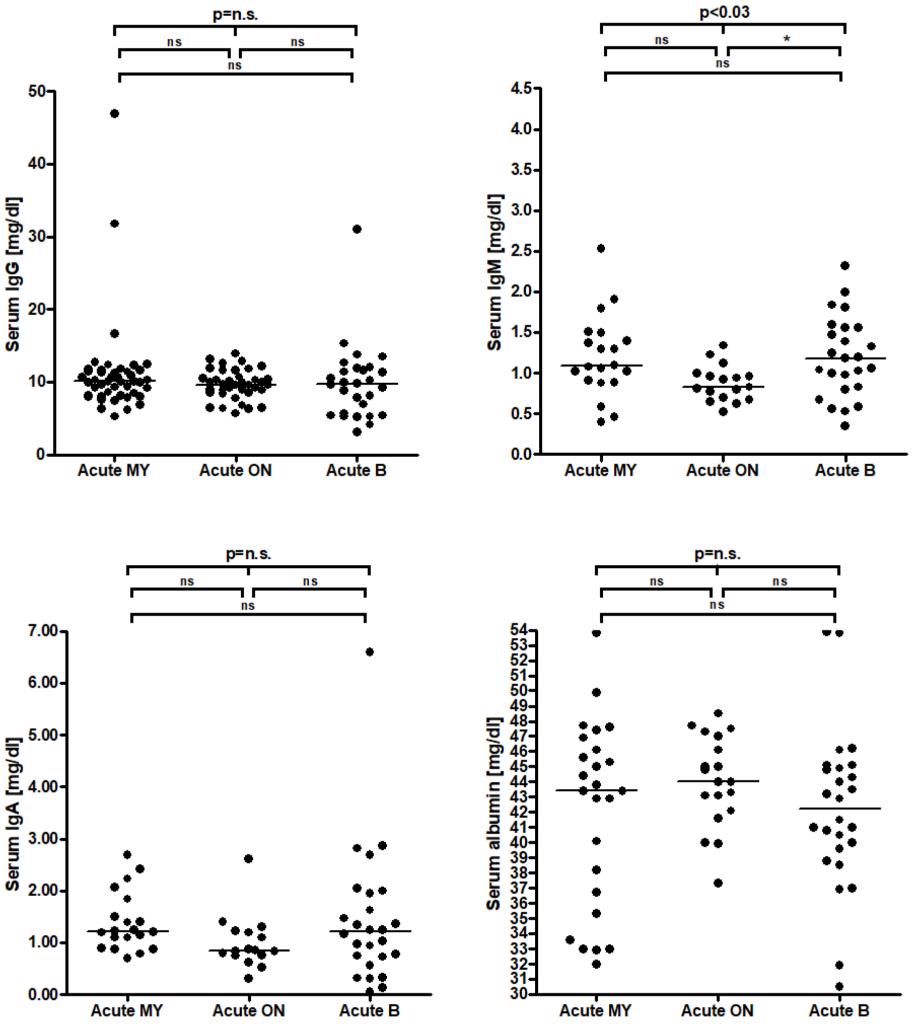

Supplementary Figure 2. No marked differences in serum IgG, IgM, IgA and albumin levels between the 'acute MY', the 'acute ON' and the 'acute BRAIN' (B) subgroup, except for slightly higher median IgM values in the 'acute BRAIN' subgroup as compared to the acute 'ON' subgroup.
