# Supplementary material for: Cerebrospinal fluid findings in patients with myelin oligodendrocyte glycoprotein (MOG) antibodies. Part 2: Results from 108 lumbar punctures in 80 pediatric patients
Source: J Neuroinflammation. 2020 Sep 3;17:262. doi: 10.1186/s12974-020-01825-1 (PMC7470445; doi:10.1186/s12974-020-01825-1)
Supplement: Supplementary file 3 — Additional file 3: Supplementary Figure 3. Regression analysis of QAlb and CSF total protein, demonstrating a close relationship between the two parameters (r2=0.75, p<0.00001). [file 12974_2020_1825_MOESM3_ESM.pdf]

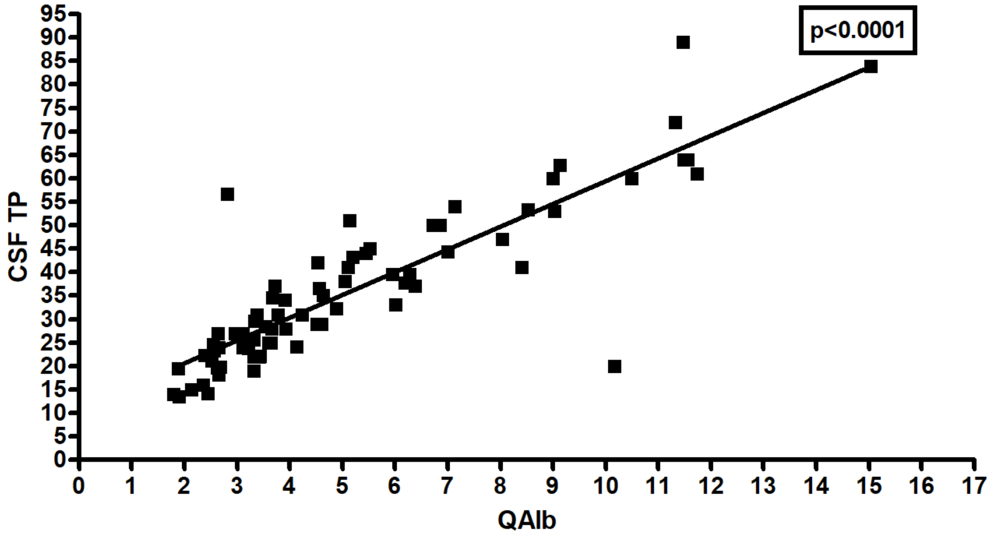

Supplementary Figure 3. Regression analysis of QAlb and CSF total protein, demonstrating a close relationship between the two parameters (r^2=0.75, p<0.00001). CSF = cerebrospinal fluid; QAlb = albumin CSF/serum ratio; TP = total protein.
