# Supplementary material for: Cerebrospinal fluid findings in patients with myelin oligodendrocyte glycoprotein (MOG) antibodies. Part 2: Results from 108 lumbar punctures in 80 pediatric patients
Source: J Neuroinflammation. 2020 Sep 3;17:262. doi: 10.1186/s12974-020-01825-1 (PMC7470445; doi:10.1186/s12974-020-01825-1)
Supplement: Supplementary file 4 — Additional file 4: Supplementary Figure 4. Influence of age at LP on CSF parameters as detected by a pooled linear regression analysis of data from the pediatric and the adult cohort (r2=0.089 for CSF TP, p<0.0001; r2=0.064 for QAlb, p=0.0004; r2=0.027 for QIgG, p<0.04; r2=0.097 for CSF L-lactate, p=0.0002). By contrast, no significant relationship was observed when the two cohort were analysed separately (not shown). [file 12974_2020_1825_MOESM4_ESM.pdf]

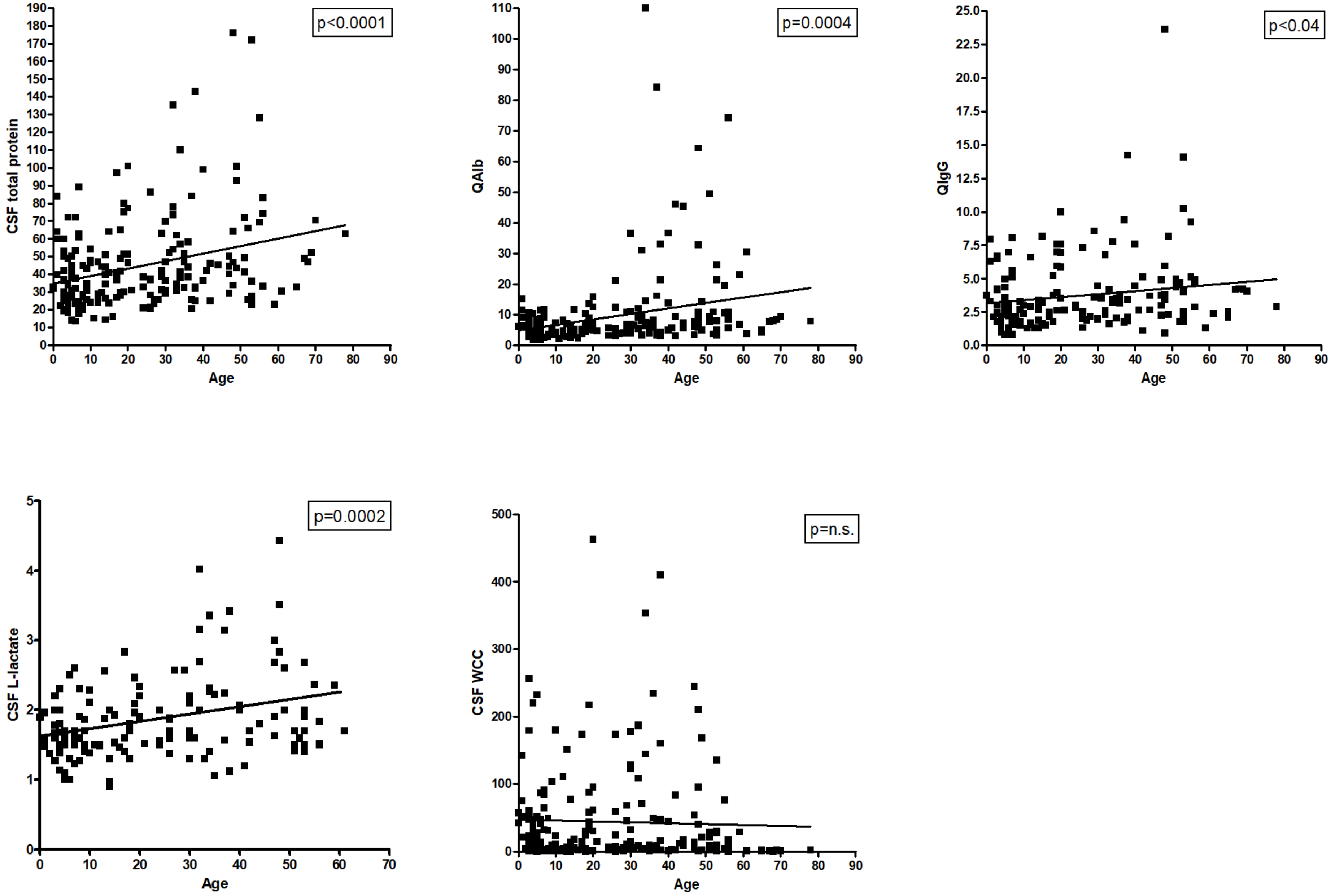
p<0.0001
CSF total protein
Age
p=0.0004
QAlb
Age
p<0.04
QIgG
Age
p=0.0002
CSF L-lactate
Age
p=n.s.
CSF WCC
Age

Supplementary Figure 4. Influence of age at LP on CSF parameters as detected by a pooled linear regression analysis of data from the pediatric and the adult cohort (r^2=0.089 for CSF TP, $p<0.0001$; r^2=0.064 for QAlb, $p=0.0004$; r^2=0.027 for QIgG, $p<0.04$; r^2=0.097 for CSF L-lactate, $p=0.0002$). By contrast, no significant relationship was observed when the two cohort were analysed separately (not shown). CSF = cerebrospinal fluid; LP = lumbar puncture; QAlb = albumin CSF/serum ratio; QIgG = IgG CSF/serum ratio; TP = total protein; WCC = white cell count.
