# Supplementary material for: Cerebrospinal fluid findings in patients with myelin oligodendrocyte glycoprotein (MOG) antibodies. Part 2: Results from 108 lumbar punctures in 80 pediatric patients
Source: J Neuroinflammation. 2020 Sep 3;17:262. doi: 10.1186/s12974-020-01825-1 (PMC7470445; doi:10.1186/s12974-020-01825-1)
Supplement: Supplementary file 5 — Additional file 5: Supplementary Table 1. CSF findings during acute attacks and during remission in the ‘acute MY’ subgroup, the ‘acute ON’ subgroup and the ‘acute BRAIN’ subgroup (stratified results from Table 10). [file 12974_2020_1825_MOESM5_ESM.pdf]

	Units	Attack MY, first LP/event	Remission MY, last LP/event	Attack ON, first LP/event	Remission ON, last LP/event	Attack BRAIN, first LP/event	Remission BRAIN, last LP/event
Pleocytosis	*samples*	22/27 (81.5%)	1/4 (25%)	5/25 (20%)	1/3 (33.3%)	18/29 (62.1%)	1/3 (33.3%)
WCC	*cells/µl*	52 (2-256;27)	4 (0-35;4)	2 (0-18;25)	1 (0-23;3)	21 (0-173;29)	4 (2-18;3)
WCC >100/µl	*samples*	9/27 (33.3%)	0/4 (0%)	0/25 (0%)	0/3 (0%)	1/29 (3.4%)	0/3 (0%)
OCB	*samples*	7/27 (25.9%)	0/2 (0%)	0/26 (0%)	0/3 (0%)	2/26 (7.7%)	0/3 (0%)
IgG-IF >10%	*samples*	3/21 (14.3%)	0/2 (0%)	0/19 (0%)	0/2 (0%)	2/20 (10%)	0/2 (0%)
QAlb > Qlim(Alb)	*samples*	14/22 (63.6%)	0/2 (0%)	5/20 (25%)	0/2 (0%)	12/22 (54.5%)	0/2 (0%)
CSF TP elevated	*samples*	10/25 (40%)	1/4 (25%)	1/24 (4.2%)	0/2 (0%)	7/28 (25%)	0/2 (0%)
CSF TP concentrations	*mg/dl*	43.6 (14-89;26)	35.85 (19.6-56.6;4)	24.86 (13.4-64;22)	25.05 (22.1-28;2)	32.2 (14.2-97.2;29)	19.5 (17-22;2)
CSF TP >100 mg/dl	*samples*	0/26 (0%)	0/4 (0%)	0/24 (0%)	0/2 (0%)	0/29 (0%)	0/2 (0%)
CSF L-lactate elevated	*samples*	10/20 (50%)	1/3 (33.3%)	3/20 (15%)	1/1 (100%)	6/19 (31.6%)	0/2 (0%)
CSF L-lactate concentrations	*mg/dl*	1.79 (1.38-2.6;20)	1.5 (1-2.11;3)	1.5 (0.97-2.56;18)	1.9 (1.9-1.9;1)	1.59 (0.9-2.83;19)	1.7 (1.69-1.7;2)
CSF L-lactate >3 mmol/l	*samples*	0/20 (0%)	0/3 (0%)	0/20 (0%)	0/1 (0%)	0/19 (0%)	0/2 (0%)
Time since attack onset	*days*	2 (0-33;28)	623 (68-1827;4)	3.5 (0-40;26)	56 (46-63;3)	1.5 (0-26;30)	48 (48-3595;3)

Supplementary Table 1. CSF findings during acute attacks (first LP/event) and during remission (last LP/event) in the ‘acute MY subgroup’, the ‘acute ON subgroup’ and the ‘acute BRAIN subgroup’ (stratified results from Table 10). CSF = cerebrospinal fluid; IgG-IF = immunoglobulin G, intrathecally produced fraction; LP = lumbar puncture; MY = myelitis; OCB = oligoclonal bands; ON = optc neuritis; TP = tital protein; WCC = white cell count.
