# Supplementary material for: Cerebrospinal fluid findings in patients with myelin oligodendrocyte glycoprotein (MOG) antibodies. Part 2: Results from 108 lumbar punctures in 80 pediatric patients
Source: J Neuroinflammation. 2020 Sep 3;17:262. doi: 10.1186/s12974-020-01825-1 (PMC7470445; doi:10.1186/s12974-020-01825-1)
Supplement: Supplementary file 6 — Additional file 6: Supplementary Table 2. Variations in OCB positivity over time. [file 12974_2020_1825_MOESM6_ESM.pdf]

	LP #1	LP #2	LP #3
Pat. #1	**OCB +/2**, M, A, QIgG neg	OCB -/1, M, Rem, QIgG neg	Not done
Pat. #2	OCB -/1, B, A, QIgG neg	**OCB +/2**, B, A, QIgG neg	Not done
Pat. #3	OCB -/4, B, A, QIgG nd	OCB -/1, B, Rem, QIgG neg	Not done
Pat. #4	OCB -/4, M, A, QIgG nd	OCB -/1, M, A, QIgG nd	OCB -/4, M, Rem, QIgG neg

Supplementary Table 2. Variations in OCB positivity over time. In 1 patient, OCB disappeared over the course of disease (absent 61 days after the initial LP following steroid treatment and during remission); in 1 patient, OCB were absent in the initial sample but were detectable in a follow-up sample taken 24 days later, respectively. Note that QIgG was normal in the OCB-positive samples, indicating low levels of intrathecal IgG synthesis. A = acute attack; B = brain/brainstem/cerebellar disease (last attack before LP); M = myelitis (last attack before LP); OCB = oligoclonal bands; ON = optic neuritis (last attack before LP); QIgG = CSF/serum immunoglobulin G ratio; Rem = remission
